# Measurement properties of patient-reported outcome measures (PROMs) used in adult patients with chronic kidney disease: A systematic review

**DOI:** 10.1371/journal.pone.0179733

**Published:** 2017-06-21

**Authors:** Olalekan Lee Aiyegbusi, Derek Kyte, Paul Cockwell, Tom Marshall, Adrian Gheorghe, Thomas Keeley, Anita Slade, Melanie Calvert

**Affiliations:** 1 Centre for Patient Reported Outcomes Research, University of Birmingham, Edgbaston, Birmingham, United Kingdom; 2 Institute of Applied Health Research, University of Birmingham, Edgbaston, Birmingham, United Kingdom; 3 Department of Renal Medicine, University Hospitals Birmingham NHS Foundation Trust, Queen Elizabeth Hospital Birmingham, Edgbaston, Birmingham, United Kingdom; 4 Oxford Policy Management Ltd, Oxford, United Kingdom; 5 PAREXEL International, London, United Kingdom; Medizinische Universitat Graz, AUSTRIA

## Abstract

**Background:**

Patient-reported outcome measures (PROMs) can provide valuable information which may assist with the care of patients with chronic kidney disease (CKD). However, given the large number of measures available, it is unclear which PROMs are suitable for use in research or clinical practice. To address this we comprehensively evaluated studies that assessed the measurement properties of PROMs in adults with CKD.

**Methods:**

Four databases were searched; reference list and citation searching of included studies was also conducted. The COnsensus-based Standards for the selection of health Measurement INstruments (COSMIN) checklist was used to appraise the methodological quality of the included studies and to inform a best evidence synthesis for each PROM.

**Results:**

The search strategy retrieved 3,702 titles/abstracts. After 288 duplicates were removed, 3,414 abstracts were screened and 71 full-text articles were retrieved for further review. Of these, 24 full-text articles were excluded as they did not meet the eligibility criteria. Following reference list and citation searching, 19 articles were retrieved bringing the total number of papers included in the final analysis to 66. There was strong evidence supporting internal consistency and moderate evidence supporting construct validity for the Kidney Disease Quality of Life-36 (KDQOL-36) in pre-dialysis patients. In the dialysis population, the KDQOL-Short Form (KDQOL-SF) had strong evidence for internal consistency and structural validity and moderate evidence for test-retest reliability and construct validity while the KDQOL-36 had moderate evidence of internal consistency, test-retest reliability and construct validity. The End Stage Renal Disease-Symptom Checklist Transplantation Module (ESRD-SCLTM) demonstrated strong evidence for internal consistency and moderate evidence for test-retest reliability, structural and construct validity in renal transplant recipients.

**Conclusions:**

We suggest considering the KDQOL-36 for use in pre-dialysis patients; the KDQOL-SF or KDQOL-36 for dialysis patients and the ESRD-SCLTM for use in transplant recipients. However, further research is required to evaluate the measurement error, structural validity, responsiveness and patient acceptability of PROMs used in CKD.

## Introduction

Chronic kidney disease (CKD) is a global health issue [1]. It affects up to 16% of the adult population in the developed world and is associated with increased morbidity and mortality that is directly related to severity [2, 3]. CKD also has major healthcare economic costs [4, 5]. For the National Health Service (NHS) in England, the total estimated expenditure attributable to CKD between 2009 and 2010 was over a billion pounds; renal replacement therapy (RRT) accounted for approximately half of this expenditure [6]. In the US, it is estimated that managing stages 3 and 4 CKD cost the Medicare fee-for-service (FFS) approximately $44.4 billion annually [7].

Early symptoms of CKD, such as fatigue, are usually non-specific [8]. However patients with more advanced CKD often report multiple ‘clusters’ of symptoms including drowsiness, pain, pruritus and dry skin [9]. This overall symptom burden may have a negative impact on the perceived health-related quality of life (HRQOL) of patients with end-stage renal disease [10, 11].

HRQOL can be assessed using self-administered, validated questionnaires known as patient-reported outcome measures (PROMs) [12]. PROMs have a wide variety of applications ranging from clinical trials [13] to product labelling [14] and routine clinical care [15, 16]. There is an increasing awareness that PROMs may have a future role in the management of patients with kidney disease, including integration into routine practice; for example, through monitoring patients for symptoms or changes in HRQOL that may require an intervention [17].

If any benefit is to be derived from the use of PROMs, it is important that they are well validated to ensure that they actually measure what they are supposed to measure, produce consistent results and capture all aspects of the construct(s) under investigation that matter to the target population if any benefit is to be derived from their use [14].

A systematic review by Gibbons and Fitzpatrick [18] evaluated the measurement properties of PROMs used in the CKD population, but this was conducted over six years ago. There have been methodological advances since then [19, 20] and it is reasonable to assume that new research has been published [21]. The review was restricted to studies published in English which might have excluded potentially relevant papers [18]. In addition, the review did not report evaluating the methodological quality of the selected studies. It is vital that the methodological quality of studies evaluating the measurement properties of PROMs is assessed to ensure that conclusions about the reliability and validity of the measures are dependable [22] as these have a potential impact on clinical practice and health policy [23].

Therefore, we have evaluated the methodological quality of the selected studies using the COnsensus-based Standards for the selection of health Measurement INstruments (COSMIN) checklist [22] and used the findings to inform our evidence synthesis. Thus providing the best evidence possible, to inform the selection of PROMs for monitoring the symptoms of CKD and its treatment effects in pre-dialysis, dialysis and renal transplant patients.

## Methods

### Design

This systematic review was conducted and reported according to a registered and published protocol (PROSPERO registration number: CRD42016035554) (See S1 Text. Review Protocol) [24] and written in compliance with the Preferred Reporting Items for Systematic Reviews and Meta-Analysis (PRISMA) guidelines (See S2 Text. PRISMA Checklist) [25]. We also considered the findings of the review of systematic reviews by Terwee et al. [26].

### Search strategy

Relevant databases including MEDLINE (Ovid), EMBASE (Ovid), PsycINFO (Ovid) and CINAHL Plus (EBSCO) were systematically searched from inception to 21^st^ December 2015 without language restrictions [24].

The search strategy was initially developed for MEDLINE and subsequently adapted for the other databases (See S3 Text. Search Strategy). Two existing search filters [27, 28] were combined with key terms generated by the review team for renal disease and its treatment modalities. An information specialist at the Institute of Applied Health Research, University of Birmingham, was consulted during the process.

Search records were downloaded into Endnote X7 and duplicates removed. In addition, the UK Renal Registry website was searched to 17^th^ May 2016.

### Screening process

All titles and abstracts were screened independently by two reviewers (OLA and TK/AG). Full-text articles were obtained for studies potentially meeting the eligibility criteria and were independently reviewed by the same reviewers. Reasons for exclusion were documented. Hand searching of reference lists and citation searching of the included papers was also conducted. At all stages, disagreements regarding eligibility were resolved through discussion and, if necessary, consultation with a third reviewer (MC/DK).

### Selection of studies

Studies were included if they: (1) focused on PROMs used specifically for measuring HRQOL and/or CKD symptoms (the constructs of interest) in any CKD population; and (2) reported either the development or evaluation of one or more psychometric properties of a PROM [24].

Articles excluded were clinical trial reports, editorials, reviews and conference abstracts. In addition, studies that focussed on clinician-assessed instruments, PROMs developed for use in patients with acute kidney injury or in patients below 18 years of age were excluded.

### Data extraction

Data from selected studies were extracted independently by two reviewers (OLA and AS) using a pre-designed data collection form and cross-checked for accuracy.

The following data were extracted where available

1. Characteristics of study populations
2. Questionnaire characteristics
3. Evidence regarding measurement properties as defined by Mokkink et al.[29] namely: reliability (test-retest reliability, internal consistency, measurement error); validity (content validity, construct validity (including hypothesis testing, structural validity and cross-cultural validity); responsiveness of questionnaires to changes over time; the setting and purpose for which the questionnaires were administered and details regarding their interpretability; operational characteristics including patient acceptability and mode and feasibility of administration; and details regarding patient involvement in the PROM development or validation process.

### Appraisal of the methodological quality of selected studies

Following the selection process, the quality of the included papers was assessed by two reviewers (OLA/AS) using a validated critical appraisal tool for studies of health measurement instruments: the COnsensus-based Standards for the selection of health Measurement INstruments (COSMIN) checklist [20, 22]. The COSMIN checklist is designed to evaluate the methodological quality of studies of psychometric properties [22]. It comprises of mini-checklists A to I (otherwise known as ‘boxes’) which correspond to each measurement property (See Table 1). Some measurement properties are named and defined differently by different authors, therefore the COSMIN definitions [29] were used to ascertain which measurement properties were evaluated by the studies. The COSMIN checklist is intended for use as a modular tool meaning that the mini-checklists (boxes) to be completed for each study will be determined by the measurement properties evaluated by the study [29]. Each mini-checklist has a set of quality items/questions which were rated individually using the COSMIN 4-point scale as 'excellent', 'good', 'fair' or 'poor'.

**Table 1:** Quality criteria for measurement properties.


Property	Rating †	Quality Criteria
**Reliability**		
Internal consistency	+	Cronbach's alpha(s) ≥ 0.70
	?	Cronbach's alpha not determined or dimensionality unknown
	-	Cronbach's alpha(s) < 0.70
Reliability	+	ICC / weighted Kappa ≥ 0.70 OR Pearson’s r ≥ 0.80
	?	Neither ICC / weighted Kappa, nor Pearson’s r determined
	-	ICC / weighted Kappa < 0.70 OR Pearson’s r < 0.80
Measurement error	+	MIC > SDC OR MIC outside the LOA
	?	MIC not defined
	-	MIC ≤ SDC OR MIC equals or inside LOA
**Validity**		
Content validity	+	All items are considered to be relevant for the construct to be measured, for the target population, and for the purpose of the measurement AND the questionnaire is considered to be comprehensive
	?	Not enough information available
	-	Not all items are considered to be relevant for the construct to be measured, for the target population, and for the purpose of the measurement OR the questionnaire is considered not to be comprehensive
Structural validity	+	Factors should explain at least 50% of the variance
	?	Explained variance not mentioned
	-	Factors explain < 50% of the variance
Hypothesis testing	+	Correlations with instruments measuring the same construct ≥ 0.50 OR at least 75% of the results are in accordance with the hypotheses AND correlations with related constructs are higher than with unrelated constructs
	?	Solely correlations determined with unrelated constructs
	-	Correlations with instruments measuring the same construct < 0.50 OR < 75% of the results are in accordance with the hypotheses OR correlations with related constructs are lower than with unrelated constructs
Cross-cultural validity	+	No differences in factor structure OR no important DIF between language versions
	?	Multiple group factor analysis not applied AND DIF not assessed
	-	Differences in factor structure OR important DIF between language versions
Criterion validity	+	Convincing arguments that gold standard is “gold” AND correlation with gold standard ≥ 0.70
	?	No convincing arguments that gold standard is “gold”
	-	Correlation with gold standard < 0.70
**Responsiveness**		
Responsiveness	+	Correlation with changes on instruments measuring the same construct ≥ 0.50 OR at least 75% of the results are in accordance with the hypotheses OR AUC ≥ 0.70 AND correlations with changes in related constructs are higher than with unrelated constructs
	?	Solely correlations determined with unrelated constructs
	-	Correlations with changes on instruments measuring the same construct < 0.50 OR < 75% of the results are in accordance with the hypotheses OR AUC < 0.70 OR correlations with changes in related constructs are lower than with unrelated constructs

MIC = minimal important change, SDC = smallest detectable change, LoA = limits of agreement

ICC = intraclass correlation coefficient, DIF = differential item functioning, AUC = area under the curve

**†** + = positive rating,? = indeterminate rating

- = negative rating

(Reproduced with permission from Caroline Terwee, COSMIN)

An item is rated ‘excellent’ when there is evidence that the methodological quality of the study in relation to the item is adequate [20]. An item is rated ‘good’ when relevant information is not reported in an article, but it can be assumed that the methodological quality is adequate [20]. An item is rated ‘fair’ if there is doubt about the adequacy of the study’s methodological quality in relation to that item [20]. Finally, an item is rated ‘poor’ when there is evidence that the methodological quality of the study in relation to that particular item is inadequate [20]. For example, a small sample size was considered poor methodological quality in all the mini-checklists. A sample size ≥100 was considered ‘excellent’, 50–99 ‘good’, 30–49 ‘fair’, and <30 ‘poor’ [20].

The 'worst score counts' method was used to determine the methodological quality of each paper per measurement property [20]. This meant taking as the overall score for each measurement property, the lowest rating given to any item within the respective mini-checklist [20].

Reviewers consulted a third author (MC/DK) if they were unable to reach a consensus at any point during the assessment.

### Data synthesis

The quality criteria developed by Terwee et al. [30] was used to rate the results for each measurement property per study as either 'positive' (+), 'indeterminate' (?) or 'negative' (-). For example, structural validity was rated as positive, if the factors identified after performing a factor analysis were reported to explain at least 50% of variance. If the factors explained <50% of variance structural validity was rated as negative. The indeterminate rating was given if the percentage of variance explained was not reported. (See Table 1) [30].

An evidence synthesis across studies was then conducted for measurement properties reported for each PROM using another set of criteria (See Table 2) [19]. At this stage, the overall level of evidence for each PROM was provided by one or more studies, taking into account their methodological quality [20]. The overall level of evidence for each measurement property was graded as ‘strong’, ‘moderate’, ‘limited’ ‘unknown’ or ‘conflicting’ [19]. For example, a measurement property was graded as ‘strong’ if at least one study had ‘excellent’ methodological quality or at least two studies had ‘good’ methodological qualities (See Table 2).

**Table 2:** Levels of evidence for the quality of the measurement property.[19, 30].


Level of Evidence	Rating^†^	Criteria
Strong	+++ or—-	Consistent findings in multiple studies of good methodological quality OR in one study of excellent methodological quality
Moderate	++ or —	Consistent findings in multiple studies of fair methodological quality OR in one study of good methodological quality
Limited	+ or -	One study of fair methodological quality
Conflicting	+/-	Conflicting findings
unknown	?	Only studies of poor methodological quality

^**†**^ + = positive rating

? = unknown rating

- = negative rating

(Reproduced with permission from Caroline Terwee, COSMIN)

## Results

**Fig 1:**
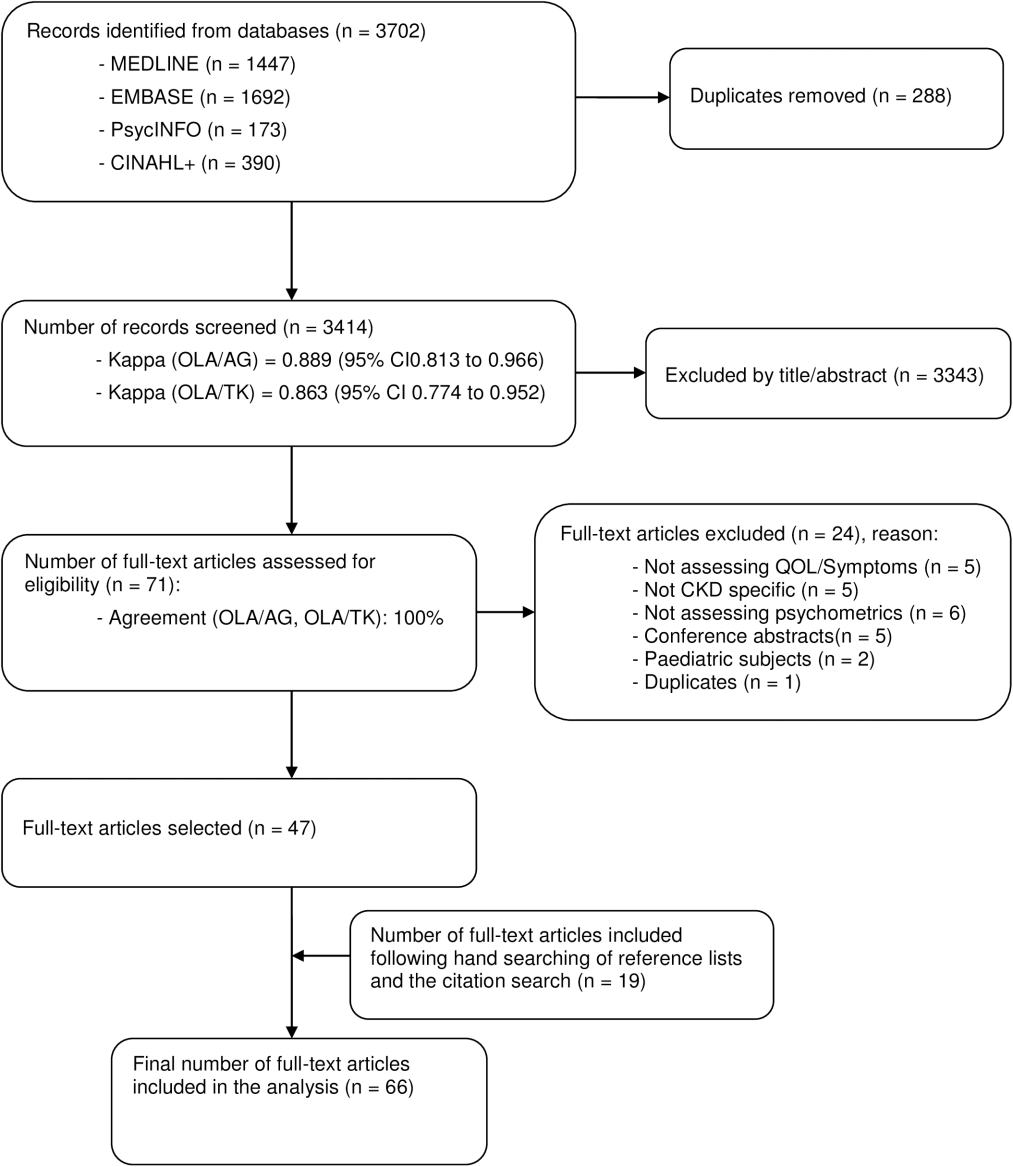
Flow diagram.

The search strategy retrieved 3,702 titles/abstracts. After 288 duplicates were removed, 3,414 abstracts were screened and 71 full-text articles were retrieved for further review. Of these, 24 full-text articles were excluded for various reasons (Fig 1). Following reference list and citation searching, 19 articles were retrieved bringing the total number of papers included in the final analysis to 66. Strength of agreement between the reviewers, calculated using Cohen’s Kappa Statistic [31], was good (OLA/AG = 0.889, OLA/TK = 0.863).

### Description of tables

Table 1 presents the quality criteria used to evaluate the measurement properties for each PROM while the criteria used for the evidence synthesis are in Table 2. A brief description of the PROMs evaluated in this review is presented in Table 3. The methodological qualities of these studies are summarized in Table 4. Table 5 presents the synthesis of the overall level of evidence for each PROM. The results reported by the included studies were extracted and are summarized in S1 Table. Summary of study results. The characteristics of the included studies are presented in S2 Table. Characteristics of included studies. The PROMs evaluated in this review are summarized in S3 Table. Characteristics of included PROMs. S1–S3 Tables have been submitted as ‘supporting information’ due to their size.

**Table 3:** Description of PROMs evaluated.


Measure	Description
Disease-specific measures: These measure health in a way that is specific to a particular disease, set of conditions, or part of the body [122].	
Agarwal	A 37-item HRQOL measure for use in non-dialysis patients with mental and physical dimensions [32]. Overall scores range from 0 to 100 with higher scores indicating better HRQOL [32].
Kidney Disease Quality of Life—36 (KDQOL-36)	A 36-item HRQOL measure designed for use in kidney disease patients undergoing dialysis. Derived from the KDQOL-SF [33]. There are 3 specific dimensions namely: symptoms and problems (ii) burden of kidney disease (iii) effects of kidney disease. It also includes two summary scales derived from the SF-12 namely: the physical (PCS) and mental (MCS) scales [34]. Overall scores range from 0 to 100 with higher scores indicating better HRQOL [33].
KDQOL-SF	An 80-item HRQOL measure designed for use in kidney disease patients undergoing dialysis [47]. Derived from the 134-item KDQOL [47, 58]. Version 1.3 differs from version 1.2 by the addition of a screening question for sexual activity [47]. There are 8 generic dimensions from the SF-36 (See below) and 8 disease-specific dimensions namely: (i) symptoms/problems (ii) effects of kidney disease on daily life (iii) burden of kidney disease (iv) work status (v) cognitive function (vi) quality of social interaction (vii) sexual function (viii) sleep. There are 3 additional dimensions namely: (i) social support (ii) dialysis staff encouragement (ii) patient satisfaction. Scores range from 0 to 100 for each dimension and higher scores indicate better HRQOL.
Chinese Dialysis Quality of Life Scale (CDQOL)	A 29-item measure designed to measure the QOL of Chinese dialysis patients. Scored on a 5-point Likert scale. Higher scores indicate better quality of life as perceived by the patient [49].
CHOICE Health Experience Questionnaire (CHEQ)	An 83-item HRQOL measure. Designed to complement the SF-36 and assess the effectiveness of dialysis modalities [50]. Comprises of 8 dimensions from SF36 (see below) combined with 7 supplementary items and 13 specific dimensions namely: (i) freedom (ii) travel restrictions (iii) cognitive functioning (iv) financial (v) restrictions on diet and fluids (vi) recreation (vii) work (viii) body image (ix) symptoms (x) sleep (xi) sexual functioning (xii) access-related problems (xiii) quality of life. An additional 2 person-specific quality of life item. Possible scores range from 0 to 100 with higher scores indicating better HRQOL [50].
Dialysis Symptom Index (DSI)	A 30-item measure designed to assess symptom prevalence and severity in patients on haemodialysis [52]. An overall symptom burden score and a total symptom severity score are calculated. Symptoms are rated on a 5-point Likert scale and total score ranges from 0 to 150 with higher scores indicating greater symptom severity [52].
Modified Edmonton Symptom Assessment System (modified ESAS)	This is a measure of symptom burden for use in dialysis patients. It is a modification of the ESAS. There are 10 symptom-specific items and 10 visual analogue scales with superimposed 0–10 scale [55]. The scale for each symptom is anchored by the words ‘No’ and ‘Severe’ at 0 and 10, respectively and the sum of scores range from 0 to 100 with higher scores indicating greater symptom distress and burden [55].
Kidney Disease Questionnaire (KDQ)	A 26-item measure designed to assess the effects of interventions on the QOL of patients undergoing haemodialysis [56]. There are 5 dimensions namely: (i) physical symptoms (ii) fatigue (iii) depression (iv) relationships with others (v) frustration. Note that the physical symptom dimension is patient-specific, thus the symptoms most important to individual patients are identified and used to evaluate the dimension. Questions are scored on a 7-point Likert scale and higher scores indicate a lower impact of disease on HRQOL [56].
KDQOL	A 134-item QOL measure designed for use in kidney disease patients undergoing dialysis [58]. It consists of SF36 dimensions (see below), 11 kidney disease targeted scales and an item that assesses change in health over a year (overall health rating) [58]. All scale scores are transformed linearly into 0–100 point scales with higher scores indicating better HRQOL [58].
KDQOL (modified)	A 55-item QOL measure derived from the KDQOL [59]. Using affinity mapping, 11 subscales [59] were identified namely: (i) pain (ii) psychological dependency (iii) cognitive functioning (iv) social functioning (v) dialysis-related symptoms (vi) cardiopulmonary symptoms (vii) sleep (viii) energy (ix) cramps (x) diet (xi) appetite. 4 items were ungrouped. The measure is scored on a 0 to 100 scale with higher scores indicating better HRQOL [59].
WHOQOL-BREF (Dialysis)	A 32-item HRQOL measure modified for use in dialysis patients with 4 domains (incorporating 4 dialysis-specific items): (i) physical (ii) psychological (iii) social relationship (iv) environment. The measure also includes two global items (general QOL and general health). A 5-point Likert scale is used and higher scores signify higher HRQOL [63].
Quality of Life Index (QLI) 3.0	A 68-item QOL measure divided into 2 sections. One section measures satisfaction with various domains of life, while the second measures the importance of the domain to the individual [64]. There are 4 domains: (i) health and functioning (ii) Social and economic (iii) psychological/spiritual (iv) family. Each section has 3 additional dialysis-related items. The total QOL score and the four subscale scores range between 0 and 30 with higher scores indicating a better HRQOL [64].
End-Stage Renal Disease Symptom Checklist-Transplantation Module (ESRD-SCL-TM)	A 43-item symptom-specific QOL measure designed for use in renal transplant patients on immunosuppression therapy [70]. There are 6 dimensions: (i) limited physical capacity (ii) limited cognitive capacity (iii) cardiac and renal dysfunction (iv) side effects of corticosteroids (v) increased growth of gum and hair (vi) transplantation-related psychological distress. A 5-point Likert scale is used. Higher scores indicate worse QOL/symptoms [70].
Modified Transplant Symptom Occurrence and Symptom Distress Scale (MTSOSD)	A 29-item measure designed to assess side effects of immunosuppression therapy in renal transplant patients [74]. The symptom occurrence dimension has 20 items while the symptom distress dimension has 9 [74]. Ridit analysis, a statistical method of analysing ordinal data [123] was chosen for data analysis [74]. Higher ridit scores indicate greater symptom distress [74].
Gastrointestinal Symptom Rating Scale (GSRS)	A 15-item measure that assesses important gastrointestinal side effects of immunosuppressive therapy in renal transplant patients [75]. There are 5 dimensions: (i) reflux (ii) diarrhoea (iii) constipation (iv) abdominal pain (v) indigestion. Each dimension gives an average score ranging from 1 (no discomfort) to 7 (very severe discomfort) with higher scores indicating worse impact [75].
Gastrointestinal Quality of Life Index (GIQLI)	A 36-item measure which focuses on the impact GI complaints on the HRQOL of renal transplant patients [75]. There are 5 dimensions: (i) GI symptoms (ii) emotional status (iii) physical function (iv) social function (v) strain of medical treatment. Total scores range from 0 to 144 with higher scores indicating a better HRQOL [75].
Kidney Transplant Questionnaire (KTQ)	A 25-item HRQOL measure for use in renal transplant patients [78]. It has 5 dimensions namely: (i) physical symptoms (ii) fatigue (iii) uncertainty/fear (iv) appearance (v) emotional. The physical symptom dimension is patient-specific, thus the symptoms most important to individual patients are identified [78]. For each dimension, an average score ranging from 1 to 7 is calculated with higher scores indicating better HRQOL in patients [78].
ReTransQoL (RTQ) version 1	A 45-item measure designed to assess QOL in renal transplant patients [80]. There are 5 dimensions: (i) physical health (ii) mental health (iii) medical care (iv) fear of losing graft (v) treatment. All dimensions are linearly transformed to a 0 to 100 scale and higher scores indicate better HRQOL [80].
ReTransQoL (RTQ) version 2	A 32-item measure designed to assess QOL in renal transplant patients [80]. There are 5 dimensions: (i) physical health (ii) social functioning (iii) medical care (iv) treatment (v) fear of losing graft. All dimensions are linearly transformed to a 0 to 100 scale and higher scores indicate better HRQOL [80].
CKD-Symptom Burden Index (CKD-SBI)	A 32-item measure of symptom burden. Derived from the DSI. The CKD-SBI was developed for use in patients with CKD stages IV and V however it was used in pre-dialysis and dialysis populations in this study [82]. The measure has 4 dimensions namely: (i) prevalence (ii) distress (iii) severity (iv) frequency. Total score ranges from 0 to 100 and higher scores indicate higher symptom burden [82].
**Generic measures: These measure health in a general manner and can be used for various health conditions [122].**	
Nottingham Health Profile (NHP)	A generic HRQOL questionnaire with 38 yes/no questions [124] grouped into 6 dimensions: (i) pain (ii) energy (iii) physical mobility (iv) sleep (v) emotional reactions (vi) social isolation. There is an optional part. The NHP scores range between 0 (good health status) and 100 (poor health status) [124].
SF-36 version 2	A generic 36-item HRQOL measure with 8 scales [125] namely: (i) physical functioning (ii) physical role (iii) bodily pain (iv) general health (v) vitality (vi) social functioning (vii) emotional role (viii) mental health. An additional 1-item measure of self-evaluated change in health status is available. The Likert rating method is used and raw scores are linearly transformed into 0 to 100 scales with higher transformed scores indicating better HRQOL [125].
SF-12	A generic 12-item HRQOL measure derived from the SF-36 [126] (see above). The 8 dimensions can be computed into 2 distinct clusters, PCS-12 and MCS-12 with higher values indicating better HRQOL [126].
**Utility measures: These provide utilities or values regarding health and can be used for cost-utility analyses of interventions [18].**	
EQ-5D	A utility measure with a self-classifier and a visual analogue scale (VAS) which can be used to value health states [127]. The self-classifier includes 5 dimensions: (i) mobility (ii) self-care (iii) usual activities (iv) pain/discomfort (v) anxiety/depression. Each dimension has 3 levels of severity (no problems, some problems, and severe problems) and it is possible to describe 243 health states between 0 (dead) and 1 (perfect health) [127].
Modified Time Trade-Off (TTO)	This utility measure was used to measure quality of life in dialysis and renal transplant populations and values range from 0 (death) to 1 (full health) [83, 128].

**Table 4:** Methodological quality of included studies.


Instrument	Study		Language				CKD population	Internal consistency	Reliability	Measurement error	Content validity	Structural validity	Hypothesis testing	Translation	Criterion validity	Responsiveness
**Agarwal**	Agarwal [32]		English				Pre-dialysis	POOR	FAIR		FAIR	POOR	FAIR			
**CDQOL**	Suet-Ching [49]		Cantonese				Dialysis	POOR	POOR		FAIR		FAIR			
**CHEQ**	Aiyasanon [51]		Thai				Dialysis	POOR					FAIR	GOOD		
	Wu [50]		English				Dialysis	POOR			GOOD		GOOD			
**CKD-SBI**	Almutary [82]		Arabic				Pre-dialysis, Dialysis	POOR					FAIR	FAIR		
**DSI**	Önsoz [53]		Turkish				Dialysis	POOR	FAIR					GOOD		
	Weisbord [52]		English				Dialysis		POOR		FAIR					
**QLI 3.0**	Dehesh [65]		Persian				Dialysis	POOR				POOR	FAIR	GOOD		
	Ferrans [64]		English				Dialysis	POOR	POOR				FAIR			
	Halabi [66]		Arabic				Dialysis	POOR						FAIR		
	Korkut [67]		Turkish				Dialysis	POOR	FAIR				FAIR	FAIR		
**KDQOL (D)**	Hays [58]		English				Dialysis	POOR			FAIR	POOR	FAIR			
**KDQOL (M)**	Rao [59]		English				Dialysis	POOR					FAIR			
**KDQOL-36**	Chao [33]		Taiwanese				Pre-dialysis	EXCELLENT				EXCELLENT	FAIR	GOOD		
	Chow [37]	Cantonese				Dialysis		POOR	POOR				GOOD			
	Mateti [42]	Kannada				Dialysis		FAIR	FAIR				FAIR	GOOD		
	Ricardo [34]	English, Spanish				Pre-dialysis		POOR					GOOD			
	Tao [38]	Mandarin				Dialysis		FAIR	POOR				FAIR			
	T'charoen [41]	Thai				Dialysis		POOR	FAIR				FAIR			
	Yang [40]	English				Dialysis		GOOD				GOOD	GOOD			
**KDQOL-SF**	Abd ElHafeez [35]	Arabic				Pre-dialysis		POOR	GOOD			POOR	FAIR	GOOD		
	Barotfi [76]	Hungarian				Transplant		POOR	FAIR				FAIR			
	Bataclan [88]	Filipino				Dialysis		POOR	FAIR				FAIR	GOOD		
	Boini [89]	French				Dialysis		POOR	FAIR				FAIR			
	Bouidida [46]	Moroccan				Dialysis		POOR	POOR				GOOD	GOOD		
	Cheung [36]	Chinese				Pre-dialysis		POOR					FAIR			POOR
	Duarte [96]	Portuguese				Dialysis			FAIR				FAIR	GOOD		
	Fardinmehr [94]	Persian				Dialysis		POOR					FAIR	GOOD		
**KDQOL-SF**	Green [85]			Japanese		Dialysis		POOR	GOOD				FAIR	GOOD		
	Hays [47]			English		Dialysis		POOR			FAIR					
	Joshi [43]			English, Mandarin, Malay		Dialysis		EXCELLENT				EXCELLENT	GOOD			
	Klersy [39]			Italian		Dialysis		POOR	POOR			POOR	FAIR	GOOD		
	Kontodimopoulos[86]			Greek		Dialysis		POOR					FAIR			
	Kontodimopoulos [87]			Greek		Dialysis		POOR					FAIR			
	Korevaar [48]			Dutch		Dialysis		POOR					GOOD	GOOD		POOR
	Malindretos [44]			Greek		Dialysis		POOR	FAIR				FAIR	GOOD		
	Molsted [92]			Danish		Dialysis		POOR						GOOD		
	Moreira [93]			Portuguese		Dialysis		POOR					FAIR			
	Pakpour [102]			Farsi		Dialysis		FAIR	POOR			FAIR	FAIR	GOOD		
	Park [45]			Korean		Dialysis		POOR	FAIR				POOR	GOOD		
	Perneger [90]			French		Dialysis		POOR					FAIR			
	Vasilieva [95]			Russian		Dialysis		POOR								
	Yildirim [91]			Turkish		Dialysis		POOR					FAIR	FAIR		
**KDQ**	Alvarez-Ude [57]			Spanish		Dialysis		POOR	FAIR				FAIR	GOOD		
	Laupacis [56]			English		Dialysis			FAIR		FAIR	POOR	FAIR			POOR
**ESRD-SCL**	Franke [70]			German		Transplant		GOOD	POOR		FAIR	GOOD	FAIR			
	Ortega [71]			Spanish		Transplant		GOOD	FAIR			GOOD	FAIR			POOR
	Stavem [72]			Norwegian		Transplant		FAIR	FAIR				GOOD	GOOD		
**GIQLI**	Kleinman [75]			German, English		Transplant		POOR					GOOD			
**GSRS**	Kleinman [75]			German, English		Transplant		POOR					GOOD			
**KTQ**	Chisholm-Burns [84]			English		Transplant		POOR					FAIR			
	Laupacis [78]			English		Transplant		POOR	POOR		FAIR	POOR	FAIR			POOR
	Niu [77]			Chinese		Transplant		GOOD	FAIR			GOOD	FAIR	GOOD		
	Rebollo [79]			Spanish		Transplant		POOR	POOR				FAIR	POOR		POOR
**ESAS**	Davison [55]			English		Dialysis			FAIR		FAIR		FAIR			
	Davison [54]				English	Dialysis										POOR
**MTSOSD**	Moons [74]				Dutch	Transplant							GOOD			
**RTQ v1**	Beauger [81]				French	Transplant						EXCELLENT				
	Gentile [80]				French	Transplant		POOR	POOR		GOOD	POOR	FAIR			POOR
**RTQ v2**	Beauger [81]				French	Transplant		GOOD				GOOD	FAIR			
**EQ-5D**	Cleemput [73]				Dutch, French	Transplant							GOOD			
**SF-12**	Pakpour [62]				Persian	Dialysis		GOOD	GOOD			GOOD	FAIR			
**SF-36 v2**	Feurer [69]				English	Transplant		POOR					FAIR			
	Mingardi [68]				Italian	Dialysis		GOOD					FAIR			
**NHP**	Badia [61]				Spanish	Dialysis		FAIR	FAIR							
	Zengin [60]				Turkish	Dialysis		FAIR					FAIR			
**TTO (modified)**	Churchill [83]				English	Dialysis, Transplant			FAIR				FAIR			
**WHOQOL-BREF (D)**	Yang [63]				Taiwanese	Dialysis		GOOD	POOR			GOOD	GOOD			

**Table 5:** Evidence synthesis of PROMs used in patients with CKD.


Instrument version	Population	Internal consistency	Reliability	Measurement error	Content validity	Structural validity	Hypothesis testing	Criterion validity	Responsiveness
**Agarwal [32]**	**Pre-dialysis**	**?**	**+**		**+**	**?**	**-**		
**KDQOL-36 [33, 34, 37, 38, 40–42]**	**Pre-dialysis**	**+++**				?*	**++**		
	**Dialysis**	**++**	**++**			?*	**++**		
**KDQOL-SF [35, 36, 39, 43–48, 76, 85–96, 102]**	**Pre-dialysis**	**?**	**++**			**?**	**++**		**?**
	**Dialysis**	**+++**	**++**		**+**	**+++**	**++**		**?**
	**Transplant**	**?**	**-**				**+**		
**CDQOL [49]**	**Dialysis**	**?**	**?**		**+**		**+**		
**CHEQ [50, 51]**	**Dialysis**	**?**			**++**		**++**		
**DSI [52, 53]**	**Dialysis**	**?**	**?**		**+**				
**ESAS [54, 55]**	**Dialysis**		**+**		**+**		**+**		**?**
**KDQ [56, 57]**	**Dialysis**	**?**	**+**		**+**	**?**	**++**		**?**
**KDQOL (D)[58]**	**Dialysis**	**?**			**+**	**?**			
**KDQOL (M)[59]**	**Dialysis**	**?**					**+**		
**NHP [60, 61]**	**Dialysis**	**++**	**?**				**+**		
**SF-12 [62]**	**Dialysis**	**++**	**++**			**+**	**+**		
**WHOQOL-BREF (D) [63]**	**Dialysis**	**++**	**?**			**?**	**-**		
**QLI 3.0 [64–67]**	**Dialysis**	**?**	**-**			**?**	**++**		
**SF-36 v2**	**Dialysis [68]**	**++**					**+**		
	**Transplant [69]**	**?**					**-**		
**ESRD-SCL [70–72]**	**Transplant**	**+++**	**++**		**+**	**++**	**++**		**?**
**EQ-5D [73]**	**Transplant**						**++**		
**GIQLI [75]**	**Transplant**	**?**					**++**		
**GSRS [75]**	**Transplant**	**?**					**++**		
**KTQ [77–79, 84]**	**Transplant**	**++**	**+**		**+**	**?**	**++**		**?**
**MTSOSD [74]**	**Transplant**						**++**		
**RTQ v1 [80, 81]**	**Transplant**	**?**	**?**		**++**	**+/-**	**+**		**?**
**RTQ v2 [81]**	**Transplant**	**++**				**++**	**+**		
**TTO (modified) [83]**	**Mixed (D & TX)**		**+**				**+**		
**CKD-SBI [82]**	**Mixed (D & Pre-D)**	**?**					**+**		

+ = positive rating

? = unknown rating

- = negative rating

+/- = conflicting findings

?* = indeterminate rating (due to non-reporting of variance explained by factors)

### Evidence synthesis

A total of 25 PROMs were identified from the 66 publications; 20 disease-specific, 3 generic and 2 utility PROMs (See Table 3). As the included studies were conducted in pre-dialysis, dialysis and renal transplant populations, the evidence synthesis is described in 3 corresponding sections (See Table 5). The term ‘indeterminate’ was used when vital information required to assess a measurement property was missing (See Table 1) while ‘unknown’ was used for measurement properties that were only assessed by studies of poor methodological quality (See Table 2).

#### Pre-dialysis population

Three disease-specific PROMs were used in pre-dialysis populations namely the: Agarwal [32], KDQOL-36 [33, 34] and KDQOL-SF [35, 36]. Although the studies in this section measured estimated glomerular filtration rates (eGFR), there was significant disparity in their description of patients at this stage of CKD. Three studies described patients as 'non-dialysis' [32], 'mild-to-moderate' CKD patients [34] and ESRD patients [36] respectively, while only the studies by Chao et al. [33] and Abd Elhafeez [35] formally categorized patients into CKD stages. None of the studies validated PROMs separately by stage of CKD pre-dialysis.

Agarwal. There was limited evidence for test-retest reliability and content validity. Internal consistency and structural validity were rated as 'unknown' [32].

Kidney Disease Quality of Life—36 (KDQOL-36). Strong evidence for internal consistency was found [33] and there was moderate evidence for hypothesis testing [33, 34]. Structural validity was rated as 'indeterminate' [33].

KDQOL-SF. There was moderate evidence for test-retest reliability [35] and hypothesis testing [35, 36]. Internal consistency, structural validity and responsiveness were rated as 'unknown' [35, 36].

#### Dialysis population

Fourteen PROMS were used in this group of patients. Among the five studies that evaluated KDQOL-36, two had mixed samples [37, 38]. As the majority of the participants in Tao et al [38] were on dialysis and no significant transplant specific symptoms were elicited in Chow et al. [37] a pragmatic decision was made to analyse them here.

Klersy et al. [39] used a sample which comprised of 85% dialysis and 15% pre-dialysis patients to assess the KDQOL-SF. Again, as the majority were dialysis patients, this study was analysed in this section.

KDQOL-36. Moderate evidence was found for internal consistency [40], test-retest reliability [41, 42] and hypothesis testing [37, 38]. However, structural validity was rated as 'indeterminate' [40].

KDQOL-SF. There was strong evidence of internal consistency and structural validity [43]. Moderate evidence was found for test-retest reliability [44, 45] and hypothesis testing [46] while there was limited evidence for content validity [47]. Responsiveness was rated as 'unknown' [48].

Chinese Dialysis Quality of Life Scale (CDQOL). Reliability (internal consistency and test-retest) for CDQOL was rated as 'unknown' and there was limited evidence for content validity and hypothesis testing [49].

CHOICE Health Experience Questionnaire (CHEQ). Moderate evidence for hypothesis testing and content validity was found [50] while internal consistency was rated as 'unknown' [50, 51].

Dialysis Symptom Index (DSI). There was limited evidence for content validity [52]. Reliability (internal consistency and test-retest) was rated as 'unknown' [52, 53].

Modified Edmonton Symptom Assessment System (modified ESAS). There was limited evidence of test-retest reliability, content validity and hypothesis testing. Responsiveness was rated as 'unknown' [54, 55].

Kidney Disease Questionnaire (KDQ). There was moderate evidence of hypothesis testing [56, 57] while there was limited evidence for test-retest reliability and content validity [56]. Structural validity, responsiveness [56] and internal consistency [57] were rated as 'unknown'.

KDQOL. There was limited evidence for content validity while internal consistency and structural validity were rated as 'unknown' [58].

KDQOL (Modified). There was limited evidence for hypothesis testing while internal consistency was rated as 'unknown' [59].

Nottingham Health Profile (NHP). There was moderate evidence for internal consistency [60, 61] and limited evidence for hypothesis testing [60]. Test-retest reliability was rated as 'unknown' [61].

SF-12. Moderate evidence was found for internal consistency and test-retest reliability. There was limited evidence for structural validity and hypothesis testing [62].

WHOQOL-BREF (Dialysis). There was moderate evidence for internal consistency while test-retest reliability and structural validity were rated as 'unknown' [63].

Quality of Life Index (QLI) 3.0. There was moderate evidence for hypothesis testing [64, 65]. Internal consistency and structural validity were rated as 'unknown' [64–67] while there was limited evidence against test-retest reliability [67].

SF-36 version 2. Moderate evidence was found for internal consistency while limited evidence was found for hypothesis testing [68].

#### Renal transplant population

Ten PROMs were evaluated specifically in renal transplant patients and all except the SF-36 and the EQ-5D were disease specific measures.

SF-36 version 2. Internal consistency was rated as 'unknown' [69].

End-Stage Renal Disease Symptom Checklist–Transplantation Module (ESRD-SCL-TM). There was strong evidence for internal consistency [70, 71], moderate evidence for test-retest reliability [71, 72], hypothesis testing [72] and structural validity [71]. There was limited evidence for content validity [70] while responsiveness was rated as 'unknown' [71].

EQ-5D. Moderate evidence for hypothesis testing was found [73].

Modified Transplant Symptom Occurrence and Symptom Distress Scale (MTSOSD). Moderate evidence for hypothesis testing was found for MTSOSD [74].

Gastrointestinal Symptom Rating Scale (GSRS). Moderate evidence for hypothesis testing was found while internal consistency was rated as 'unknown' [75].

Gastrointestinal Quality of Life Index (GIQLI). Moderate evidence for hypothesis testing was found while internal consistency was rated as 'unknown' [75].

KDQOL-SF. There was limited evidence for hypothesis testing and limited evidence against test-retest reliability. Internal consistency was rated as 'unknown' [76].

Kidney Transplant Questionnaire (KTQ). There was moderate evidence for internal consistency [77] and hypothesis testing [78, 79]. Limited evidence was found for test-retest reliability [77] and content validity [78] Structural validity [77] and responsiveness [79] were rated as 'unknown'.

ReTransQoL (RTQ) version 1. There was moderate evidence of content validity [80], conflicting evidence of structural validity [80, 81] and limited evidence of hypothesis testing [80]. Internal consistency, test-retest reliability and responsiveness were rated as 'unknown' [80].

RTQ version 2. This revised version had moderate evidence for internal consistency and structural validity. There was still limited evidence for hypothesis testing [81].

#### Single studies with mixed samples

The study by Almutary et al. [82] evaluated the CKD-Symptom Burden Index (CKD-SBI) in a mixed sample of pre-dialysis and dialysis patients while Churchill et al. [83] assessed the Time Trade-Off (TTO) in a mixed sample of dialysis and renal transplant recipients.

CKD-Symptom Burden Index (CKD-SBI)

Limited evidence was found for hypothesis testing while internal consistency was rated as 'unknown' [82].

Modified Time Trade-Off (TTO)

There was limited evidence for test-retest reliability and hypothesis testing [83].

### Other findings

#### Reliability

Internal consistency: Of the 58 studies that assessed internal consistency, 42 were scored 'poor' for methodological quality. This was due to one of 3 reasons:

1. Some studies did not conduct a factor analysis and did not reference a relevant study that conducted one [41, 49–51, 53, 61, 64, 75, 82].
2. Some studies conducted a factor analysis but had inadequate sample sizes by COSMIN standards [32, 35, 39, 47, 59, 65, 78, 80].
3. Some studies referenced a relevant study that conducted a factor analysis but the sample size used for the study was inadequate by COSMIN standards [34, 36, 37, 44–48, 57, 66, 67, 69, 76, 79, 84–95].

Test-retest reliability: Thirty-four studies conducted test-retest reliability and most of them reported internal correlation coefficients (ICC). The study by Duarte et al. [96] was the only one that reported inter & intra-observer reliability. The majority scored 'fair' for test-retest reliability and this was largely due to the small sample sizes.

Measurement error: The included studies did not provide adequate information on parameters such as the minimal important change (MIC) [97], the standard error of measurement (SEM) [30, 98] or the limits of agreement (LOA) [99] making it difficult to assess measurement error. Only one study provided an estimate for minimal clinical important difference (MCID) [75].

#### Validity

Content validity: This was assessed by development studies for RTQ [80], modified ESAS [55], Agarwal [32], CHEQ [50], CDQOL [49], DSI [52], KDQ [56], KTQ [78], KDQOL [58], KDQOL-SF [47]. Six validation studies reported content validity indexes (CVI) [33, 38, 49, 53, 77, 82] and of these only Suet-Ching [49] reported patient involvement in the process of content validation.

Construct validity (Hypothesis testing): Of the 66 studies evaluated, only 13 reported clearly formulated a priori hypotheses or expectations regarding the magnitude and direction of correlations. For this reason, most of the studies were rated as 'fair' for the methodological quality of their hypothesis testing.

The absence of clear a priori hypotheses make it difficult to determine whether any results reported for construct validity and responsiveness was due to chance or not [100, 101].

Structural validity: A number of measures were rated poorly for structural validity due to issues with factor analysis (See internal consistency). Out of the 19 studies that conducted a factor analysis, 8 were scored 'poor' [32, 35, 39, 56, 58, 65, 78, 80] and this can be attributed to the use of sample sizes inadequate by COSMIN standards (n < 5 times the total number of items and < 100) [100]. It is important that studies perform factor analysis as it verifies scale structure and uni-dimensionality which determines the scoring and interpretation of a measure’s internal consistency statistic [100].

Criterion validity: This was not assessed for any study as the COSMIN Delphi panel does not regard any PROM as true 'gold standard' [29] and the FDA holds a similar view [14].

Cross-cultural validity/Translations: Twenty-five studies translated PROM instruments and adapted them to varying degrees for their study population. As none of these studies performed a multi-group confirmatory factor analysis or assessed differential item functioning (DIF) between language groups, the decision was made not to assess cross-cultural validity. Therefore, only the quality of their translations was assessed according to the provisions specified in the COSMIN manual [100].

The translations conducted by 20 studies [33, 35, 39, 42, 44–46, 48, 51, 53, 57, 65, 72, 77, 85, 88, 92, 94, 96, 102] were rated as good, while the translations by 4 studies [66, 67, 82, 91] were rated as fair and 1 translation was rated as poor [79].

#### Responsiveness

Responsiveness was only assessed by 8 of the included studies [36, 48, 54, 56, 71, 78–80] and all were rated ‘poor’ as the information provided was inadequate.

#### Interpretability

A number of the PROMs included in this review had significant floor and ceiling effects which might indicate a reduction in their ability to discriminate between patients with the lowest or highest possible scores and detect changes over time [30].

Ten instruments (KDQOL-SF, Agarwal, KDQ, KDQOL-36, KTQ, QLI, KDQOL, ESRD-SCL, CHEQ and NHP) had floor and ceiling effects > 15% while the RTQ v2 and SF-12 [62, 81] had < 15%.

The ‘work status’ dimension of the KDQOL-SF had a 70% floor effect [45, 86] while the ‘social support’, ‘patient satisfaction’ and ‘staff encouragement’ domains had ceiling effects of 46%, 45% and 67% [86], respectively. Sexual function had ceiling effects of 53.3% while cognitive function had ceiling effects of 60% [35]. The ‘pain’ dimension of the SF-36 had ceiling effects as high as 59% [35].

Eight studies namely; RTQ v1 [80], RTQv2 [81], GSRS & GIQLI [75], KDQOL-36 [38, 41], KDQOL-SF [44], CHEQ [51], TTO [83] reported measurement scores for subgroups within their study populations. While some of the differences in scores were statistically significant, it is unclear if any were clinically relevant.

#### Feasibility and acceptability

This was difficult to assess for the included studies as less than a third reported the average time needed to complete the questionnaires, few reported the recall period used and none reported administrative requirements for collection and analysis of data. However, most of the studies reported good response rates which suggest that patients might find the use of PROMs acceptable.

The fact that majority of the studies failed to report the level and/or the method used for handling missing data meant there might be a risk of bias [101]. Questions relating to sexual activities had the highest levels of missing data with Bataclan and Dial [88] reporting a response rate of <18% in Filipino patients [88].

#### Patient involvement

Patient involvement in the process of item generation and/or item selection was reported in the development of the RTQ [80], KDQ [56], KTQ [78], ESRD-SCLTM [70], KDQOL-SF [47], Agarwal [32], CHEQ [50], QLI 3.0 [64], KDQOL (Dialysis) [58] and CDQOL [49].

All the studies that translated and adapted measures reported pre-testing their translations in patients to assess a combination of comprehension, cultural relevance and acceptability except Rebollo et al.[79]

## Discussion

This is the first review to use the COSMIN checklist [100] to evaluate the measurement properties of PROMs used in patients with CKD. In all, 25 PROMs were evaluated by a total of 66 studies in pre-dialysis, dialysis, and renal transplant patients.

In the pre-dialysis population, the KDQOL-36 exhibited strong evidence for internal consistency and moderate evidence for construct validity (hypothesis testing). It should be noted that the evidence for this measure was obtained from studies conducted in Taiwanese patients [33], and a combination of Hispanic and non-Hispanic white patients [34]. Furthermore, the measurement properties were not reported by CKD stage. Therefore, further validation may be necessary before use outside these study populations and/or where focus is on the use of PROMs in relationship to the severity of CKD.

In dialysis patients, we found evidence to support the use of both the KDQOL-SF and the KDQOL-36. The KDQOL-SF demonstrated strong evidence for internal consistency and structural validity and moderate evidence for test-retest reliability and construct validity (hypothesis testing) while the KDQOL-36 had moderate evidence for internal consistency, test-retest reliability and construct validity (hypothesis testing). Again it should be noted that this evidence was obtained from a significant number of non-English studies, further validation work would be needed before these measures could be confidently utilised in an English speaking population.

In renal transplant patients, the ESRD-SCLTM demonstrated strong evidence for internal consistency and moderate evidence for test-retest reliability, structural validity and construct validity (hypothesis testing).

Consistent with the review by Gibbons and Fitzpatrick [18], much of the evidence we present was derived from cross-sectional studies. However, in contrast to that study, we did not include clinical trial reports as the methodological quality of their PROM evaluations are often unsatisfactory [23] and their PROM analysis often inadequate and insufficiently [103] reported for any meaningful evaluation to be possible [19].

In line with our findings, Gibbons and Fitzpatrick [18] found evidence to support the use of the KDQOL-SF but did not specify which modality of renal replacement therapy (RRT) provided the evidence. We found evidence to support the use of the KDQOL-36 (which was not available at the time Gibbons and Fitzpatrick [18] conducted their review), and the ESRD-SCLTM (which was excluded from their study) [18]. The ERA-EDTA expert panel [104] recommended the KDQOL-36, following a consensus meeting.

There were methodological issues with the majority of the PROMs included in this review. These included: sample sizes smaller than current recommendations, a lack of clearly described a priori hypotheses and inadequate reporting of missing data, and little or no information on measurement error and responsiveness. Similar issues were reported by Gibbons and Fitzpatrick [18] and highlighted in reviews for other health conditions [105–107].

Given that the COSMIN standards only became available within the last decade, it is unsurprising that most of the earlier studies fared poorly when judged against these exacting methodological and reporting standards even though they might meet the minimum standards recommended by the International Society for Quality of Life Research (ISOQOL) [108]. However, this highlights the need to test and revise PROM instruments on a regular basis to ensure they actually perform as intended according to contemporary psychometric standards.

This systematic review provides a basis for identifying PROMs with potential utility in clinical practice. There is evidence that the use of PROMs in clinical practice could enhance communication [109–112] between patients and their clinicians. Basch et al. [113] noted that the use of PROMs in routine clinical care, could facilitate the reporting of serious adverse events due to drug toxicities [113], while a review by Finkelstein et al. [114] suggested that the use of PROMs could assist renal teams with the development of strategies to improve the HRQOL of the patient with CKD [114]. Calvert et al. [115] suggested that PROM data could potentially facilitate the delivery of tailored healthcare if successfully integrated with routinely collected clinical and laboratory data [115].

Whilst effective management of risk factors can slow CKD progression [116] many patients with severe pre-dialysis CKD progress to end stage renal disease (ESRD) and in this group PROMs may have a significant role [117–119]. For example, PROMs could be used to monitor individual patients for symptoms or changes in HRQOL that may indicate that a medical review or intervention is needed [17, 120].

Whilst a systematic review can provide valuable evidence on psychometric properties of PROMs, clinicians and researchers need to consider a number of issues such as the domains covered by different measures, the available supporting evidence, and the suitability for the target population and use (whether for clinical trials, routine practice, audit or real-time decision making). As no single measure covers all the domains that might be of interest, there might be a need to administer more than one measure. Patient acceptability is also a key issue. Therefore, it is important that patients are involved not just in the development of measures but also in the selection for research and/or practice.

During the course of this review, we became aware of the existence of the IPOS-Renal and contacted its developers [121]. This measure is currently being used within the measurement work stream of the UK Renal Registry and its validation by the Palliative care Outcome Scale (POS) team is on-going [121]. Therefore, evidence to support its use may be available in future.

The key strengths of this review are the use of the COSMIN standards and criteria for evidence synthesis which ensured that our assessments of the included PROMs were robust, the stratification of the review by stage of CKD and the absence of language restrictions which strengthened our findings. The main limitation is the fact that the included studies did not adequately report their assessments for a number of measurement properties thus making it difficult and sometimes impossible to evaluate these properties.

At present, we suggest the use of the KDQOL-36 in pre-dialysis patients though initial validation might be required. We recommend the KDQOL-SF and the KDQOL-36 for use in dialysis patients as we found evidence supporting both of these measures. The shorter 36-item KDQOL-36 may be more practical for use in routine clinic settings, while the longer 80-item KDQOL-SF might be preferred for research purposes where more detailed information may be required. We suggest using the ESRD-SCLTM in renal transplant recipients to assess issues pertaining to renal transplantation and immunosuppression therapy. These measures are recommended based on the fact that they currently possess the best evidence available according to COSMIN standards in these populations and meet the minimum standards recommended by ISOQOL [108]. However, it must be recognised that none of these measures possess evidence underpinning *all* measurement properties and some lack validation in English-speaking populations. Future work should be undertaken to address these gaps. For all measures, it is vital that content validity is established according to FDA guidelines to ensure that they actually measure the concept(s) of interest. This should be conducted before other measurement properties are fully evaluated and adequately reported in order to facilitate their subsequent evaluation. Investigators may use this review to identify the gaps in evidence and design studies to address these issues in future.

## Supporting information

S1 Text Review protocol. (PDF)

S2 Text PRISMA checklist. (DOC)

S3 Text Search strategy. (PDF)

S1 Table Summary of study results. (DOCX)

S2 Table Characteristics of included studies. (DOCX)

S3 Table Characteristics of included PROMs. (DOCX)
