# Supplementary material for: Measurement properties of patient-reported outcome measures (PROMs) used in adult patients with chronic kidney disease: A systematic review
Source: PLoS One. 2017 Jun 21;12(6):e0179733. doi: 10.1371/journal.pone.0179733 (PMC5479575; doi:10.1371/journal.pone.0179733)
Supplement: S1 Table — (DOCX) [file pone.0179733.s004.docx]

| **S1 Table. Summary of study results** | | | | | | |
| --- | --- | --- | --- | --- | --- | --- |
| **Measure** (Study) | **Measurement properties** | | | | | |
|  | **Internal consistency** | **Reliability** (Sample size, time interval) | **Hypothesis testing** | **Structural validity** | **Content validity** | **Responsiveness** |
| **Agarwal 2010**  (Unnamed) [32] | Cronbach’s alpha, 4 factors: > 0.85 | ICC: 0.75 - 0.85  (n = 41, 8 weeks) | Regression, NP score vs MHC score(KDQOL) r^2^: 0.41 (P<0.001) | CFA (Varimax rotation)  KMO: 0.835.  73% of the total variance explained by factors. | Literature review and patient interviews | _ |
| **Chinese Dialysis Quality of Life Scale (CDQOL)**  Suet-Ching 2001 [49] | Cronbach's alpha (overall): 0.85 | Pearson r: 0.92, p<0.02  (n = 10, 2 weeks) | CDQOL vs WHO-BREF, Pearson r : +0.79, p = 0.000  Item-total correlations: 0.22 - 0.55 | _ | CVI: 0.94  Face validity with patients | _ |
| **CHOICE Heath Experience Questionnaire (CHEQ)**  Aiyasanon et al. 2009 [51] | Cronbach's alpha: >0.70 for all domains (except 'social functioning', 0.66 and 'quality of life', 0.58). | _ | Significant differences between patients on PD and HD in 10 domains with patients on HD scoring higher.  Patients with DM scored significantly lower than those without DM in 5 domains. | _ | _ | _ |
| Wu et al. 2001 [50] | Cronbach's alpha: >0.70 for all domains (except quality of life 0.68). | _ | Item-scale correlations at least two SEs greater than item to other scales.  Patients with ESRD and other co-morbidities had significantly lower physical functioning scores (p < 0.001) than those without these conditions. | _ | Literature review and patient focus groups | _ |
| **CKD Symptom Burden Index (CKD-SBI)**  Almutary et al. 2015 [82] | Cronbach’s alpha: 0.91 - 0.93 | _ | Pearson's coefficient Inter-correlations between domains,0.82 - 0.97  Pearson’s correlations between all symptom domains and each domain. r = -0.33 (prevalence vs burden of KD) to r = -0.78 (distress vs symptom scale).(p<0.01) | _ | CVI overall: 0.98  Item range: 0.88 - 1.00 | _ |
| **Dialysis Symptom Index (DSI)**  Önsoz and Yesilbalkan 2013 [53] | Cronbach's alpha for symptoms: 0.82 - 0.84 | Kappa statistics: 0.10 - 0.89 (n = 120, 1 week) 23 items kappa values ≥ 0.60  Diarrhoea and difficulty concentrating had values < 0.20 | _ | _ | Expert rating of items, CVI: 0.82 | _ |
| Weisbord et al. 2004 [52] | _ | Kappa statistics: 0.06 - 0.90 (n = 20, 4 -7 days) 10 items demonstrated kappa values >0.60. | _ | _ | Expert review and focus groups with patients and providers. | _ |
| **Ferrans and Powers Quality of Life Index 3.0 (Dialysis)**  Dehesh et al. 2014 [65] | Cronbach's alpha: 0.64 - 0.88 | _ | Spearman's correlation between items and hypothesised domains: 0.75 - 0.94 (scaling success 100%) | Factor analysis (Quartimax rotation): Variance explained by the four factors was 89%. | _ | _ |
| Ferrans and Powers 1985 [64] | Cronbach's alpha: 0.90 | Test-retest: 0.81  (n = 20, 4 weeks) | Correlation between scores from the QLI and the life satisfaction question for dialysis patients = 0.65. | _ | Literature review and patient consultation | _ |
| Halabi 2006 [66] | Cronbach's alpha: 0.79 - 0.86 | _ | _ | _ | _ | _ |
| Korkut 2007 [67] | Cronbach's alpha: 0.92 | Test-retest: 0.65 (n = 31, 4 weeks) | Correlations between QLI and 6 SF-36 domains (r = 0.36 - 0.47, p < 0.05)  Correlations between QLI score and the Beck's score (r = -0.566, p<0.01) | _ | _ | _ |
| **Kidney Disease Quality of Life(KDQOL Dialysis version)**  Hays 1994 [58] | Cronbach's alpha: all >0.75 (except quality of social interaction 0.68) | _ | Number of medications currently taken correlated significantly with 9 domains with physical functioning being the highest (r = -0.23, p <0.01) | CFA (oblique Promax rotation)  Guttman's weakest lower bound was consistent for 3 to 4 factors. 4 factors were indicated by parallel analysis and the scree test. | Literature review and patient focus groups | _ |
| **KDQOL (Modified)**  Rao et al 2000 [59] | Cronbach's alpha: 0.66 - 0.92 | _ | Correlation of pain domain with corresponding SF36 pain domain, r = 0.70, p<0.001  Sleep and SF36 energy/fatigue domains, r = 0.57, p<0.001 | _ | _ | _ |
| **KDQOL-36 questionnaire (KDQOL-36)**  Chao et al. 2016 [33] | Cronbach's alpha (excluding item 28): 0.76 - 0.92 | _ | Correlation between item to total and item to domain: 0.21 - 0.67 and 0.43 - 0.92 respectively (p< .001).  Pearson’s Coefficient correlation of the KDQOL-36 (excluding Item 28) to the question “How satisfied are you with your health?” of the WHOQOL-BREF): 0.28 to 0.42 (p < 0.001) | After improving the model fit (deleting item 28 (dialysis) and item 35 (sex life), the modified model 2 was presented as follows: χ2 = 1390.903, χ2/df = 2.711, p < 0.001, GFI = 0.833, adjusted GFI = 0.806, RMSEA = 0.063. | The equivalence, clarity, and readability of the translated KDQOL-36™ CVI: 91%, 93%, and 95%, respectively. | _ |
| Chow and Tam 2014 [37] | Cronbach's alpha (kidney disease domains): 0.65 - 0.83. Cronbach's PCS: 0.32 and MCS: 0.53. | Test-retest (ICC) all subscales: >0.98.  (n = 20, 10 - 14 days) | Spearman Rho correlations depression (HADS) and all domains, r: −0.25 (p < 0.001) to −0.50 (p < 0.001).  Independent t-test, to compare QoL between the patient groups, p <0.001 for all domains. | _ | _ | _ |
| Mateti 2015 [42] | Cronbach's alpha: 0.72 - 0.77 | Test-retest (ICC): 0.83 - 0.97 (n = 45, 7 days) | EQ-VAS in kidney disease targeted domains correlated significantly with symptoms (0.393), effects of kidney disease (0.320) and burden of kidney disease (0.264). | _ | _ | _ |
| Ricardo et al. 2013 [34] | Cronbach’s alpha: 0.80 - 0.87 | _ | MCS correlated with BDI for all groups: -0.56 to -0.61, p<0.001.  The PSF score correlated significantly with the symptoms domain, r= −0.70 to −0.77. | _ | _ | _ |
| Tao et al. 2014 [38] | Cronbach’s alpha: 0.69 - 0.78 | Test-retest (ICC): 0.70 -0.86(n = 28, 10 - 14 days) | Spearman’s correlations between all KDQOL-36 scores and the BDI score, rho: -0.395 to -0.654, p <0.01) | _ | Item-level content validity index (I-CVI) and scale-level CVI (S-CVI): 1.0 | _ |
| Thaweethamcharoen et al. 2013 [41] | Cronbach's alpha, kidney disease domains:  HD = 0.79 - 0.83  PD = 0.71 - 0.78 | Test-retest (ICCs): for all scales > 0.95 (n = 30) | For HD, Spearman's correlations of disease specific scores with PCS, MCS and utility scores (EQ5D): all +ve and significant.  For PD, Spearman's correlations of symptoms domain: all +ve and significant (p<0.001) | _ | _ | _ |
| Yang 2013 [40] | Cronbach’s alpha, kidney disease domains:0.82- 0.91 Cronbach’s alpha:0.74 (PCS) and 0.70 (MCS) | _ | Pearson's Coefficient, item-to-scale correlation: 0.76 - 0.90 | CFA* (kidney disease items), a 3-factor model reflected the grouping of items to form the 3 kidney scales. Good fit to data. (CFI 0.934, RMSEA 0.085). | _ | _ |
| **KDQOL-Short Form questionnaire (KDQOL-SF)**  Abd ElHafeez et al. 2012 [35] | Cronbach’s alpha, kidney-specific domains: >0.70 (except 'work status' 'cognitive function', 'quality of social interaction' 0.28, 0.60. 0.23, respectively) Cronbach’s alpha, generic domains: >0.70 | Test-retest (ICC): 0.79 - 0.95  (n = 50, 1 week) | All the disease specific domains correlated significantly with PCS, r: 0.24 - 0.68 (p<0.05)  All the disease specific domains, except 'sexual function', correlated significantly with MCS, r:0.24 - 0.55 (p<0.05) | EFA (Varimax rotation)  KMO: 0.73. Bartlett’s test of sphericity: Χ2 = 1.83, p < 0.001.  PCA was done. Initial eigenvalues showed that 39 items explained 70.9% of the variance in 10 components. Factor loading ranged from 0.50 to 0.94. | _ | _ |
| Barotfi et al. 2006 [76] | Cronbach’s alpha, HD, all generic domains: > 0.70 Disease-specific: 0.54 - 0.83  Cronbach’s alpha, Tx, all generic domains: > 0.70 Disease-specific: 0.59 - 0.95 | Test–retest (Pearson's coefficients)  Disease-specific domains: 0.40 – 0.88  Generic domains: 0.56 – 0.81 (n = 63) | Differences in mean scores between Tx and HD for all generic domains and for 7 of the 11 disease-specific domains. >10 points for most domains that show a difference (p<0.001).  QoL scores significantly different between Tx and HD for generic domains. | _ | _ | _ |
| Bataclan and Dial 2009 [88] | Cronbach's alpha, Disease-specific domains: 0.60 - 0.78 Generic domains: 0.61 - 0.80 | Test-retest (ICC) :  Disease-specific domains: 0.58 - 0.97  Generic: 0.65 - 0.98  (n = 30, 10 - 14 days) | All the items in generic domains had significant correlation with overall health rating: 0.61 - 0.80, (p<0.05) except for role – emotional (p = 0.19).  Disease-specific domains correlated with the overall health rating: 0.18 – 0.39, p <0.05 | _ | _ | _ |
| Boini et al. 2007 [89] | Cronbach's alpha, Disease-specific domains: >0.7 for all except 'work status' (0.38) and 'quality of social interactions' (0.48).  Generic domains: all >0.75 except 'Social functioning' (0.64 | Test-retest correlation coefficient, specific domains: >0.70 for all except 'social interactions' (0.51), 'social support' (0.42)  Generic domains: >0.70 for all except 'physical functioning' (0.56) and 'role physical' (0.67)  (7 days) | Item-dimension correlations, for all the specific domains: 0.03 - 0.83  Correlation between burden of kidney disease (specific) and general health (generic), r = 0.72 | _ | _ | _ |
| Bouidida et al. 2014 [46] | Cronbach's alpha, Kidney-specific domains: 0.38 - 0.90. Note that sexual function only had 40% response rate.  Generic domains: 0.70 - 0.86 | Test-retest (ICC),  Kidney specific domains: 0.78 - 0.90  Generic domains: 0.67 - 0.90  (n = 20, 10 - 14 days) | Correlations of items within domains were higher than that of items outside domains in 87% of cases.  Men scored significantly higher than women on 5 subscales: on symptom/problem, cognitive function, physical functioning, social functioning, and pain. | _ | _ | _ |
| Cheung et al. 2012 [36] | Cronbach’s alpha for specific ranged from 0.32 (work status) to 1.00 (dialysis staff encouragement and sexual function)  Cronbach’s for RAND 36 domains ranged: 0.78 - 0.98 | _ | Spearman’s rank coefficient (rho), KPS scores most strongly correlated with 'effects of kidney disease' (rho = 0.56, p<0.001), 'physical functioning' (rho = 0.87, p<0.001), and 'energy/fatigue' (rho = 0.62, p<0.001).  eGFR correlated with 'burden of kidney disease' (rho = 0.32, p<0.01), 'effects of kidney disease' (rho = 0.29, p<0.01), 'symptoms/problems' (rho = 0.22, p<0.01). | _ | _ | Starting RRT was associated with improvement in the following domains: symptoms/problems (p <0.001), cognitive function (p =0.047), and sleep (p =0.044). |
| Duarte et al. 2005 [96] | _ | Intra-observer reliability (Cronbach's alpha): all >0.70 except 'patient satisfaction' (0.65).  (ICC): 0.49 - 0.93, p<0.001  Inter-observer reliability (Cronbach's alpha): all >0.70 except 'patient satisfaction' (0.50).  (ICC): 0.33 - 0.99, p<0.001 | Pearson's correlation coefficients, physical functioning and physical capacity (NHP): 0.72, p<0.05.  Energy/fatigue and Fatigue (KDQ): 0.68, p<0.05. | _ | _ | _ |
| Fardinmehr et al. 2012 [94] | Cronbach's alpha: 0.85 | _ | Pearson's coefficients correlations, Overall health score correlated significantly with Kidney specific domains: symptoms/problems (0.47), effect of KD (0.67), burden of KD (0.43). | _ | _ | _ |
| Green et al. 2001 [85] | Cronbach's alpha,  Specific (before item deletion): 0.35 - 0.92  (after deletion): 0.64 - 0.81  Generic domains: 0.73 - 0.92 | Test and retest (ICC), Specific domains: 0.42 - 0.95  Generic domains: 0.60 - 0.83  (n = 60, 10 - 14 days) | 11 out of 42 items correlated with their intended scale within 2 standard errors of their correlation with at least one other scale. | _ | _ | _ |
| Hays et al. 1995 [47] | Cronbach's alpha: all were >0.80 except quality of social interaction (0.61) and cognitive function (0.68) | _ | _ | _ | Literature review and focus groups with patients | _ |
| Joshi et al., 2010 [43] | Cronbach's alpha: Overall: >0.7  All domains: >0.7 except social function (0.66) | _ | Significant correlations between overall health score and kidney-disease targeted domains: 0.21 - 0.37 | EFA (Varimax rotation) Total variance explained by all 8 subscales was 68.35%. Physical function explained the highest percentage of variance at 31.39% | _ | _ |
| Klersy et al. 2007 [39] | Cronbach's alpha:  Kidney specific domains: Only 3 domains were <0.70.  Generic domains: Only 2 domains were <0.70 | Test-retest, Pearson's correlation coefficient and ICC ranged from 80% to 100% for all domains except staff encouragement and work status (both 60%).  (n = 10, within 4 weeks) | Item internal consistency was 74.5% for kidney specific items and 87.5% for generic items. | Factor analysis (PCA, oblique rotation, scree plot)  Eight scales related to the burden of the disease, were loaded on the first axis while scales social support, staff encouragement and patient satisfaction, were loaded on the second axis. | _ | _ |
| Kontodimopoulos & Niakas 2005 [86] | Cronbach's alpha >0.7 for all domains except social interaction, (0.59) and social support, (0.68). |  | Item-scale correlations (Item-internal consistency) for both generic and specific domains: 0.31 - 0.94. Scaling success (>0.40) in all except one generic item and 3 specific items.  T-test and ANOVA showed statistically significant differences due to age, gender, education, co-morbidities and hospitalizations (p<0.05). |  |  |  |
| Kontodimopoulos & Niakas 2007 [87] | Cronbach's alpha: >0.75 for all generic domains.  Specific domains: >0.75 for all except social interaction (0.59), Social support (0.67) | Item-internal consistency between items and their hypothesised scales for both generic and specific sections: 0.32 - 0.93. | Pearson's correlations between generic and specific domains: 0.27 - 0.67 (p<0.01).  T-test and ANOVA showed statistically significant differences due to age, gender, education, co-morbidities and hospitalizations. |  |  |  |
| Korevaar et al. 2002 [48] | Cronbach's alpha, specific domains: >0.7 for all except 'work status' and 'quality of social interactions' (both 0.39) and 'social support' (0.67). | _ | EuroQoL overall health rating (VAS-score) was significantly correlated with all the generic and specific domains of KDQOL: 0.31 - 0.76, except 'patient satisfaction'.  Residual GFR correlated significantly with 5 out the 8 specific domains, 0.15 - 0.21 | _ | _ | Significant correlations between changes in HRQOL scores and residual GFR in 10 domains within a 9 month period. |
| Malindretos 2010 [44] | Cronbach's alpha: >0.90 for all domains. | Test-retest (ICC): 0.88 - 0.99  (n = 50, within 4 weeks) | All items showed significant correlation with their hypothesized scale and this correlation was stronger than the correlation with other domains. | _ | _ | _ |
| Molsted et al. 2005 [92] | Cronbach’s alpha: > 0.70 for 8 disease-specific domains.  Cronbach’s alpha: Generic domains: 0.77 - 0.93 | _ | _ | _ | _ | _ |
| Moreira et al. 2009 [93] | Cronbach's alpha, Specific domains: 0.1 -1.0  Generic domains: 0.7 - 0.9 | _ | Ordinal logistic regression,  There were significant correlations between men and 'pain'. Men had higher scores in this dimension compared to women. | _ | _ | _ |
| Pakpour et al. 2011a [102] | Cronbach’s alpha, Specific domains: 0.71 - 0.92  Generic domains: 0.73 - 0.93 | Test–retest (ICC),  Specific domains: 0.79 - 0.92.  Generic domains: 0.77 - 0.91  (n = 20, within 10 days) | Pearson's Correlations,  Overall health rating correlated significantly with symptoms, effects, burden of kidney disease, work status, quality of social interaction, sexual function and sleep (0.11 - 0.439, P < 0.01). | PCA on the 43 kidney targeted items. KMO: 0.71, Bartlett’s test of sphericity: x2-test (946) = 4.570, P < 0.001.  EFA on the 43 items: initial eigenvalues showed all items explained 79.81% of the variance in 11 components. | _ | _ |
| Park et al. 2007 [45] | Cronbach's alpha,  Disease specific domains: 0.45 - 0.93  Generic domains : 0.65 - 0.92 | Test-retest, (ICC):  >0.75 for all KDQOL-SF domains, except social functioning (0.69)  (n = 30, 1 week) | Pearson's correlation coefficients, overall health rating correlated significantly with symptoms (0.35), effects of KD (0.35), burden of KD (0.29), cognitive function (0.22), and quality of social interaction (0.25). | _ | _ | _ |
| Perneger 2003 [90] | Cronbach's alpha,  Generic domains: 0.74 - 0.90  Disease-specific: 8 domains were >0.70. | _ | Six kidney disease specific scales correlated significantly with PCS: 0.29 (work status) to 0.56 (symptoms/problems)  All the disease specific scales correlated significantly with MCS: 0.27 (work status) to 0.70 (cognitive function). | _ | _ | _ |
| Vasilieva 2007 [95] | Cronbach's alpha: 0.72 to 0.86 (for 7 of the kidney specific domains) | _ | _ | _ | _ | _ |
| Yildirim et al. 2007 [91] | Cronbach's alpha: 0.84 | Test-retest (Cronbach’s alpha): 0.91. | Pearson's coefficient,  Significant correlations between the scores of similar domains of 15D and KDQOL-SF. Physical functioning and mobility (r = -0.81), emotional well-being and mental function (r = -0.78), sexual function and sexual activity ( r = -0.78), patient satisfaction and distress (r = -0.80). | _ | _ | _ |
| **Kidney Disease Questionnaire (KDQ)**  Alvarez-Ude et al. 1997 [57] | Cronbach's alpha: All >0.7 except relationship with others (0.68) and frustration (0.67) Overall: 0.93 | Test-retest (ICC): All >0.7 except frustration (0.62)  (n = 34, 2 weeks) | Spearman's rank correlation coefficient,  KDQ correlated with the domains and overall score of the SIP, r: -0.45 to -0.83. Correlation with the EuroQoL tariff values: 0.39 - 0.65. | _ | _ | _ |
| Laupacis et al. 1992 [56] | _ | Test-retest (ICC) placebo-treated patients: All domains >0.80  (8 weeks) | The physical and fatigue domains correlated significantly with all the other measures (Physical SIP, Psychosocial SIP, Time trade-off, Stress test) | Factor analysis was done to assist with item selection but details not reported. | Literature review, clinician and patient interviews | Significant improvement over 6 months with erythropoietin treatment in 4 of 5 dimensions: physical, fatigue, relationship with others and depression. |
| **ESRD Symptom Checklist–Transplantation Module (ESRD-SCL)**  Franke et al. 1999 [70] | Cronbach's alpha: 0.76 - 0.85 | Reproducibility (ICC) between the first and second measurement for 88 stable patients after 12 months were moderate in 5 and modest in 1 of the 6 scales. | All the domains of the ESRD-SCL correlated negatively and significantly with all the domains of SF-36 except the 'increased growth of gum' and hair' dimension. | Factor analysis (Varimax rotation) was used to create the final questionnaire. Six factors were defined which contained 43 items. | Literature review, clinician and patient interviews | _ |
| Ortega et al. 2007 [71] | Cronbach’s alpha: 0.92 (overall)  Range: 0.62 - 0.86 | Test-retest (ICC): all domains >0.7.  Range: 0.73 - 0.81 | Correlations between the ESRD-SCL and MCS: moderate for psycho-neurological problems, Limited Physical Capacity, and Limited Cognitive Capacity and weak for the rest. | Factor analysis (Varimax rotation) Scree test showed that the 6 factor model explained 56.07% variance. | _ | The ESRD-SCL scores decreased throughout follow up while the scores for SF-36 and EQ5D increased, indicating better HRQoL. |
| Stavem and Ganss 2006 [72] | Cronbach's alpha: 0.72 - 0.81  (overall: 0.94) | Test-retest (ICC): 0.87 - 0.95  (n = 48, over 2 weeks) | Pearson's coefficients,  Overall ESRD-SCL correlated negatively and significantly with all SF-36 domains. 'Limited physical capacity' correlated negatively with all SF-36 domains (-0.52 to -0.74, p<0.01). | _ | _ | _ |
| **Gastrointestinal Quality of Life Index (GIQLI)**  Kleinman et al. 2006 [75] | Cronbach's alpha, GIQLI for domains: 0.71 - 0.90  Overall: 0.93 | _ | Pearson's correlations, GIQLI total score and EQ-5D: r = 0.63.  GSRS scores were significantly different by clinical severity ratings from 'none' to 'severe and very severe'. | _ | _ | _ |
| **Gastrointestinal Symptom Rating Scale (GSRS)**  Kleinman et al. 2006 [75] | Cronbach's alpha, GSRS: 0.71 - 0.91 (except 'abdominal pain' 0.58) | _ | Pearson's correlations, 'abdominal pain' and EQ-5D: r = -0.54  GSRS scores were significantly different by clinical severity ratings from 'none' to 'severe and very severe'. | _ | _ | _ |
| **Kidney Transplant Questionnaire (KTQ)**  Chisholm-Burns et al. 2011 [84] | Cronbach's alpha: 0.62 - 0.90. | _ | Pearson's correlations, PCS (SF-12) with uncertainty/fear (0.2), physical, fatigue (0.4).  MCS with uncertainty/fear (0.3), emotional, fatigue (0.4), Physical (0.26), appearance (0.28). | _ | _ | _ |
| Laupacis et al. 1993 [78] | Cronbach's alpha: 0.61 - 0.94 | ICC: 0.70 - 0.87  (n = 26 stable Tx patients, assessed 6 and 12 months post-transplantation) | The physical domains of the SIP correlated most closely with the physical symptoms and fatigue domains of the KTQ (p < 0.001). | A combination of factor analysis and clinical judgement was used to create the final questionnaire. Details were not reported. | Literature review, clinician and patient interviews | Significant improvement in all KTQ domains before and 6 months post-transplantation, except appearance. |
| Niu et al. 2015 [77] | Cronbach's alpha: 0.70 - 0.90 | Test-retest (Pearson's coefficient): 0.70 - 0.91  (2 weeks) | Significant positive correlations with KTQ scores were obtained for PCS and MCS.  There were higher correlation co-efficients between the MCS and the KTQ domains (0.34-0.62). | CFA* analysis, (GFI 0.903), RMSEA (0.001), CFI (1.00) | Expert consultation. CVI: >0.80 | _ |
| Rebollo et al. 2003 [79] | Cronbach's Alpha: > 0.80 for all except 'Appearance' (0.69). | ICC: > 0.80 for all domains except, 'Physical symptoms' (0.63) and 'Appearance' (0.67). ICC was calculated in 'stable' patients between months 6 and 12 after Tx. | Correlations among the dimensions of the KTQ: 0.32 - 0.72.  Correlation coefficient between MCS and fatigue was 0.60. MCS and Energy was 0.78. | _ | _ | For the 'physical symptoms' domain of the KTQ the effect size at 12^th^ month was 0.48 |
| **Modified Edmonton symptom assessment system (ESAS)**  Davison et al. 2006a [55] | _ | Test-retest (ICC),  Individual symptoms: 0.53 - 0.71.  Total symptom distress score: 0.70  (n = 165, 1 week) | Pearson's Coefficient, all ESAS items significantly correlated with HRQOL scores of KDQOL-SF.  Total symptom distress score with PHC (r = -0.54, p<0.01) with MHC (r = -0.62, p<0.001) | _ | Expert review and patient participation | _ |
| Davison et al. 2006b [54] | _ | _ | _ | _ | _ | Change in overall symptom distress score correlated with changes in disease-specific (KDQOL-SF) domains, r = -0.46 to -0.73, p<0.01. |
| **Modified Transplant Symptom Occurrence and Symptom Distress Scale (MTSOSD)**  Moons et al. 2001 [74] | _ | _ | Pearson’s correlation coefficient between the ridits of symptom occurrence and symptom distress: 0.54, p=0.0001.  Positive correlations between depression and the ridits of symptom occurrence (r=0.32, p=0.001), and symptom distress (r=0.54, p<0.0001) indicating a higher level of symptom experience in depressed people. | _ | Literature review Multidisciplinary expert review | _ |
| **ReTransQoL (RTQ) Version 1**  Beauger et al. 2013 [81] | _ | _ | _ | PCA (Varimax rotation) showed an unsatisfactory structure of the items with the dimensions with a KMO of 0.841.  The 5 dimensions were confirmed with a variance of about 50%. | _ | _ |
| Gentile et al. 2008 [80] | Cronbach's alpha: 0.70 - 0.86 | _ | Physical health correlated with all domains of SF36. Significant correlation with PHC and MHC: >0.60.  Mental health correlated with all related domains of the SF36 (>0.60). Significant correlation with MHC: >0.76. | Factor analysis (Varimax rotation). Five factors identified which accounted for 46.3% of the total variance. Nine out of 45 items had for their specific dimension a factor loading under 0.40. | Tx patients confirmed the pertinence of the five dimensions, and the relevance of the items. | Significant differences were neither found for the five domains of RTQ nor domains of SF36 among any of these groups of patients. |
| **ReTransQoL (RTQ) Version 2**  Beauger et al. 2013 [81] | Cronbach's alpha: 0.7 - 0.9. | _ | The physical health domain showed high correlations with domains of SF36 namely: Vitality, General Health, Physical Functioning and Bodily Pain (r >0.65).  Women had lower HRQOL scores in comparison with men in every domain (p<0.05). | PCA (Varimax rotation): The structure of the 5 factors was fixed and supported by PCA and the 5 factors accounted for 53.1% of total variance.  The structure of the RTQ V2.0 was established with a CFA* providing satisfactory indicators: RMSEA = 0.05, SRMR = 0.049, CFI = 0.97 and GFI = 0.91. | _ | _ |
| **EuroQol EQ-5D**  Cleemput et al. 2004 [73] | _ | _ | Chi-square test show older patients have more mobility problems than younger patients (p<0.01) and have lower EQ-5D index scores (Kruskal-Wallis test, p = 0.01).  Spearman rank correlation coefficients between EQ-5D index scores and the SF-36 domain: 0.41 - 0.68 (P<0.01). | _ | _ | _ |
| **Medical Outcomes Study Short Form-12 Questionnaire**  Pakpour et al. 2011b [62] | Cronbach’s alpha,  PCS (SF-12): 0.89  MCS (SF-12): 0.90 | Teat-retest: 0.67 (bodily pain) - 0.90 (physical functioning).  All >0.7 except bodily pain.  PCS: 0.76  MCS: 0.74  (n = 70, weeks) | Patients that were female, older, less educated, dialysed for longer, and had lower levels of albumin and Kt/V had significantly (p< 0.05) poorer quality of life compared to other patient subgroups.  All items showed higher correlation when compared with their own scale than when compared with other scales. | CFA was done on the 8 scales loaded with the two latent factors (PCS and MCS). The model provided a good fit for the data, with a fit index of chi2 = 23.30, degree of freedom = 13, P = 0.04, GFI = 0.96, adjusted GFI = 0.90, normed fit index = 0.99, CFI = 0.99, standardized root mean square residual = 0.036, and RMSEA = 0.079. | _ | _ |
| **Medical Outcomes Study 36-item Short Form Health Survery (SF- 36) v 2.0**  Feurer et al. 2004 [69] | Cronbach's alpha, PCS: 0.76  MCS: 0.80 | _ | Correlation between functional performance (KPS) and PCS: 0.38, p<0.001. | _ | _ | _ |
| Mingardi et al. 1999 [68] | Cronbach's alpha: 0.70 - 0.94 | _ | Correlation between items and hypothesized scale, overlap corrected): 0.32 - 0.82. Scaling success: 100% for all except general health (80%).  Significant but low associations between SF-36 domains and albumin values | _ | _ | _ |
| **Nottingham Health Profile (NHP)**  Badia et al. 1994 [61] | Cronbach's alpha: 0.58 - 0.85  Overall: 0.91 | Test-retest, Spearman's coefficient: 0.69 - 0.85. Agreement percentage (AP): 0.48 - 0.65  Overall AP: 0.56 | _ | _ | _ | _ |
| Zengin et al. 2014 [60] | Cronbach's alpha  NHP: 0.73 - 0.97 | _ | Spearman's correlations for similar domains of NHP and SF-36: 0.33 - 0.76 | _ | _ | _ |
| **TIME TRADE-OFF (modified TTO)**  Churchill et al. 1987 [83] | _ | Test-retest, (ICC):  Overall = 0.81 (n = 171, 4 - 8 weeks) | TTO mean scores and Patient VAS scores,  Tx patients scored the highest on both scales 0.84 and 0.86 respectively. | _ | _ | _ |
| **WHOQOL-BREF (Dialysis Module)**  Yang et al. 2006 [63] | Cronbach's alpha: 0.72 - 0.79 | Test-retest: 0.61 - 0.79  (n = 20, 4 - 8 weeks) | Pearson's correlation coefficients between the 4 domains and the WHOQOL-BREF global items were less than 0.50 (p < 0.005).  Correlations between items and their corresponding domains were significant (p < 0.001) and greater than those between the items and other domains. | EFA identified 4 factors: general non-social, socioeconomic, physical-environment and physico-psychological factors.  CFI (0.93), NNFI (0.92)  All factor loadings for the items were significant | _ | _ |

^a^ The quality of translations carried out by studies was evaluated as cross-cultural validity was not assessed.

ICC - Internal correlation coefficients, NP - Neuropsychiatric, MHC - Mental health composite, R - Regression, EFA - Exploratory factor analysis, CFA - Common factor analysis, CFA* - Confirmatory factor analysis, SE - Standard error, CVI - Content validity index, PCS - Physical composite summary score, MCS - mental composite summary score, KDCS - Kidney disease composite summary, PCA – Principal component analysis, KMO - Kaiser-Meyer-Olkin, GFI - Goodness-of-fit index, RMSEA - Root mean square error of approximation, CFI - Comparative fit index, BMI - Body mass index, GFR - Glomerular filtration rate

CAD - coronary artery disease, DM – Diabetes, HD – Haemodialysis, PD – Peritoneal dialysis, Tx – Renal transplant, RRT - Renal replacement therapy, QoL – Quality of life BDI - Beck depression inventory, KPS - Karnofsky performance scale, SIP - Sickness impact profile, PSF - Patient Symptom Form
