# Supplementary material for: Measurement properties of patient-reported outcome measures (PROMs) used in adult patients with chronic kidney disease: A systematic review
Source: PLoS One. 2017 Jun 21;12(6):e0179733. doi: 10.1371/journal.pone.0179733 (PMC5479575; doi:10.1371/journal.pone.0179733)
Supplement: S2 Table — (DOCX) [file pone.0179733.s005.docx]

| **S2 Table. Characteristics of included studies** | | | | | | |
| --- | --- | --- | --- | --- | --- | --- |
| **Study** | **Type of study** | **Study population and setting** | **Country** | **Sample size**  (% of females) | **Mean age in years**  (SD or range) | **Duration of condition/treatment**  Months, Mean (SD) unless otherwise stated |
| Abd ElHafeez et al., 2012 [35] | Adaption, reliability and validation study of pre-dialysis CKD patients | Randomly selected pre-dialysis CKD patients (stages 1 – 4) from an out-patient nephrology clinic. | Egypt | 100 (46.0) | Median: 54  (42-60) | Not reported |
| Agarwal 2010 [32] | Development and validation study in non-dialysed patients with CKD | Purpose was to measure the presence and severity of symptoms.  Patients with pre-dialysis CKD attending renal clinics. CKD stages were not specified. | USA | 92 (5.0) | 67.5 (+11.0) | Not reported |
| Aiyasanon et al., 2009 [51] | Validation and reliability study | Patients attended the outpatient nephrology clinic, chronic PD clinic and chronic HD unit. | Thailand | Main study  110 (49.1) | 55.6 | HD or PD > 6 |
| Almutary et al., 2015 [82] | Developmental study based on the DSI instrument | The measure was developed to assess symptoms in different stages of CKD. A convenience sample of CKD patients from three hospitals. The majority of the patients used for the study were on dialysis while <25% were pre-dialysis (CKD stages 4 & 5). | Saudi Arabia | 433 (47.1) | 48.2 (± 14.9) | Not reported |
| Alvarez-Ude et al., 1997 [57] | Cultural adaptation and validation study in HD patients. | Patients receiving long-term HD (> 3 months) from a General Hospital were enrolled for the study. | Spain | 35 (43.0) | 67.7 (+ 10.9) | CRF: 49.6 (+ 61.8) years |
| Badia et al., 1994 [61] | Validation study in ESRD patients | Patients from a Hospital HD programme. | Spain | At follow up  121 (42.4) | 53.0 (+13.0) | HD: 48.0 (+ 35.0) |
| Barotfi et al., 2006 [76] | Translation and validation study Hungarian version of KDQOL-SF | Patients on maintenance HD at 9 dialysis units and patients from an outpatient transplant centre. | Hungary | HD 418 (44.0)  Tx 418 (41.0) | HD 53.0 (+14.0)  Tx 49.0 (+12.0) | Maintenance dialysis >3 |
| Bataclan and Dial  2009 [88] | Cultural adaptation and validation study in dialysis patients | Patients with CRD, undergoing renal replacement therapy at a dialysis centre | Philippines | 80 (56.0) | 53 (+2.0) | Not reported |
| Beauger et al., 2013 [81] | Phase 1 - Re-evaluation and adaptation study  Phase 2 & 3 – Validation study in Tx patients | Patients were Tx recipients. Phase 1 and phase 2 samples were obtained from a cross-sectional multicentre study.  Phase 3 sample came from a cross-sectional multicentre study (QUAVIREIN: French translation of Renal Quality of Life study). | France | Phase 1 & 2 1059 (38.1)  Phase 3 1591 (39.5) | 55.2 (+12.4) (Phase 1 &2)  55.3 (+14.2) (Phase 3, QUAVIREIN) | Not reported |
| Boini et al., 2007 [89] | Translation and validation study in dialysis patients | Dialysis patients from 5 centres in France | France | 68 (45.6) | 66.0 (+15.2) | Renal failure:  3.9 (± 3.7) years |
| Bouidida et al., 2014 [46] | Cultural adaptation and validation study | Hospital patients on HD and PD. | Morocco | 80 (70.0) | 43.9 (+ 14.2) | Dialysis  104.8 (+ 120) |
| Chao et al., 2016 [33] | Validation study in patients with CKD 1 - 5 | Participants were patients with CKD stages 1 - 5 from 3 CKD clinics. | Taiwan | 428 (41.0) | 62.0 (+12.3) | Not reported |
| Cheung et al., 2012 [36] | Translational and validation study in ESRD patients | This analysis is part of a larger study on the QOL and role of palliative care in ESRD (CKD stage 5).  Participants were from renal wards and outpatient clinics of a tertiary hospital. A proportion later commences dialysis. | Singapore | 78 (44.9) | Median 76 (52 - 98) | Not reported |
| Chisholm-Burns et al., 2011 [84] | Validation study of KTQ | Renal transplant recipients enrolled in an RCT, on immunosuppressant therapy. | USA | 100 (48.0) | 51.0 (+13.2) | Post-Tx  39.7 (± 29.8) |
| Chow and Tam 2014 [37] | Validation and reliability study of Cantonese Chinese version of KDQOL-36 | Patients attending the renal dialysis unit of a regional hospital and its satellite dialysis centre. | China | D 110 (31.8)  Tx 122 (44.3) | D 58.2 (+15.2)  Tx 51.8 (+10.3) | Dialysis > 3  Tx> 1 year |
| Churchill et al., 1987 [83] | Cross-sectional study of modified Time Trade-Off (TTO) | ESRD patients from regional hospital nephrology programme (dialysis and transplant patients) | Canada | 171 (?) | ? | >3months |
| Cleemput et al., 2004 [73] | Validation study | The secondary data analyses of a study of Tx patients following an immunosuppressive regimen. Tx patients from 3 centres. | Belgium, Netherlands | 361 (40.3) | 51.7 (+ 13.0) | Median post-Tx: 16.7  (Q1 = 7.9; Q3 = 38.6) |
| Davison et al., 2006a [55] | Validation study of modified ESAS in dialysis patients | Peritoneal dialysis patients and in-centre and HD patients from satellite units. | Canada | 507 (47.3) | 63.5 (+16.0) | Dialysis:  3.4 (+2.8) years |
| Davison et al., 2006b [54] | Validation study of a modified ESAS in HD patients | Longitudinal study of HD patients, in-centre and from satellite units.. | Canada | At follow up  261 (43.7) | At follow up  64.1 (+15.9) | Dialysis:  3.8 (+ 3.2) years |
| Dehesh et al., 2014 [65] | Validation study in HD patients | HD patients from a dialysis centre and two hospitals. | Shiraz, Iran | 150 (41.0) | 50.5 (+15.1) | HD > 6 |
| Duarte et al., 2005 [96] | Cultural adaptation and validation study in ESRD patients | Random sample of ESRD patients on chronic dialysis from a dialysis unit. | Sao Paulo, Brazil | Reliability test 93 (45.0) Validity test sample 74 | 49.0 (+13.0) | Median time ESRD 2 years |
| Fardinmehr et al., 2012 [94] | Reliability and validation study | ESRD patients undergoing regular HD at hospitals. | Iran | 50 (34.0) | 52.7 (+ 4.2) | Dialysis:  37.8 (+ 36.0) |
| Ferrans and Powers 1985 [64] | Developmental study of instrument for use in healthy and ill individuals | The QLI was developed to measure QOL of healthy individuals, as well as those on dialysis. | USA | Patients  37 (28) | 50 (+14.18) | Not reported |
| Feurer et al., 2004 [69] | Re-validation study in Tx patients | Clinics for solid organ transplant candidates and recipients. Data for this study was collected during an earlier study exploring HRQOL after solid organ transplantation. | USA | 112 (39.0) | 44.0 (+1.3) | Not reported |
| Franke et al., 1999 [70] | Developmental study | The ESRD SCL-TM was developed to assess the QOL of Tx recipients with a focus on side effects of immunosuppression therapy. The study was conducted during regular outpatient visits. | Germany | 458 (45.0) | 48 (+ 13.0) | Tx: 77.6 (+ 51.6) |
| Gentile et al., 2008 [80] | Development and validation study in Tx patients | Tx recipients were randomly selected from the registry of a transplant centre. | France | Validation phase  130 (36.5) | Validation phase  49.4 (± 12.8) | Validation phase  5.4 (+ 3.1) years |
| Green et al., 2001 [85] | Cultural adaptation and validation study in HD patients | Patients from dialysis centres | Japan | 712 (793?)  unknown (39.1?) | 55 | Dialysis > 3 |
| Halabi 2006 [66] | Translation, cultural adaptation and validation study | Dialysis patients |  | 270 (?) | ? | Not reported |
| Hays et al., 1994 [58] | Developmental study | Patients on dialysis at 9 outpatient dialysis centres. | USA | 165 (52.0) | 53  (22 - 87) | 78% on dialysis at the same centre >1 year 1 month. |
| Hays et al., 1995 [47] | Developmental study | The KDQOL Short Form was developed as a short version of the original KDQOL instrument. | USA | 165 (?) | Not reported | Not reported |
| Joshi et al., 2010 [43] | Validation study | HD patients at 22 dialysis centres. | Singapore | 'Evaluable' sample 980  (43.9) | 'Evaluable' sample  56 (+ 21) | HD > 3 |
| Kleinman et al., 2006 [75] | Validation study | Post-Tx patients on immunosuppression therapy attending 5 clinics in 4 countries. | Australia, Canada, Germany, and Switzerland. | 96 (43.7) | 47.4 (+ 12.3) | Tx: 3.6 (+ 3.6) years |
| Klersy et al., 2007 [39] | Cultural adaptation and validation study | A convenience sample of pre-dialysis (CKD stage not specified) and dialysis patients was enrolled at 2 referral centres. Some of the participants were hospitalized patients. | Italy | 188 (37.0) | 60.0 (+ 16.0) | Dialysis (IQR):  1.7 (0.8-5.4) years |
| Kontodimopoulos & Niakas 2005 [86] | Reliability and validation study | ESRD patients in 20 dialysis units | Greece | 483 (38.8) | 59.9 (+14.6) | HD: 6.9 (± 5.7) years |
| Kontodimopoulos & Niakas 2007 [87] | Reliability and Validation study | ESRD patients were recruited from 25 dialysis units. | Greece | 642 (38.7) | 58.1 (±14.9) | Dialysis:  6.2 (± 5.7) years |
| Korevaar et al., 2002 [48] | Validation study of disease-specific scales only | Multi-centre prospective study in 32 centres, the Netherlands Cooperative Study on the Adequacy of Dialysis (NECOSAD). | Netherlands | 375 (39.0) | 60.0 (+16.0) | Hospitalization in last 3 months:  8.8 (+ 15.0) days |
| Korkut 2007 [67] | Translational, Reliability and validation study | HD patients at a dialysis centre | Turkey | 31 (48.4) | 45.7 (+13.5) | Dialysis:  5.1 (1-12) years |
| Laupacis et al., 1992 [56] | Developmental study | The questionnaire was developed for use in clinical trials in patients on chronic HD. Stable HD patients from 3 hospitals which provide in-hospital HD were recruited. | Canada | 50 (40.0) | 55.0 (+ 15.0) | Dialysis:  41 (+ 2.0) |
| Laupacis et al., 1993 [78] | Developmental study | The KTQ was assessed in an on-going prospective cohort study of patients before and after renal transplantation at two university centres. | Canada | Development sample:  50 (28.0)  Psychometric study sample:  73 (?) | Development sample:  43 (19 - 65) | Development sample (Post-Tx): 1.3 (0.2 - 3.0) years  Psychometric sample (Post-Tx): < 6 |
| Malindretos 2010 [44] | Cultural adaptation and validation study. | Randomly selected patients undergoing HD in 6 Renal Units. | Greece | 200 (45.0) | 62.9 (+ 14.7) | HD: 47.3 (+ 57.9) |
| Mateti 2015 [42] | Cross-cultural adaptation. reliability and validation study | Patients were selected randomly from out-patient HD units from three centres. | Indian | 82 (28.1) | 50.7(± 12.3) | HD: 38.1(± 25.3) |
| Mingardi et al., 1999 [68] | Validation study | This study was part of a large, multicentre trial (DIA-QOL).  Patients were treated at 4 dialysis units. | Italy | 246 (48.2) | 60.8 (+13.1) | Not reported |
| Molsted et al., 2005 [92] | Reliability study | HD and PD patients from 2 hospitals were recruited. | Denmark | 130 (33.0) | 59.0 | HD: 39 (+ 59)  PD: 27 (+ 26) |
| Moons et al., 2001 [74] | Translation and Validation study | Post Tx out-patients of a university hospital on a maintenance immunosuppression. | Belgium | 108 (39) | 47.0 | Median post-Tx 5.5 years |
| Moreira et al., 2009 [93] | Reliability and validation study | CRF patients on HD treatment at a clinic. | Brazil | 147 (48.3) | 51.8 (+ 14.2) | HD:28.6 (+ 30.5) |
| Niu et al., 2015 [77] | Cultural adaptation, Reliability and validation study | Tx recipients from a follow-up clinic. | China | 136 (27.9) | 43.9(+ 11.4) | Tx: 40.4(+ 32.9) |
| Önsoz and Yesilbalkan 2013 [53] | Cultural adaptation and reliability study | Chronic HD patients from two dialysis units. | Turkey | 120 (44.2) | 54.5 (+ 13.8) | HD: 48.1 (41.2) |
| Ortega et al., 2007 [71] | Validation and reliability study | To validate for routine use in clinical practice. Patients were recruited from Tx waiting list at 16 hospitals. | Spain | 307 (40.8) | 51.6 (+12) | Not reported |
| Pakpour et al., 2011a [102] | Cultural adaptation and validation study | Randomly sampled patients who had been referred to dialysis centres. | Iran | 212 (43.8) | 57.5 (+ 14.7) | Dialysis:  47.9 (+ 30.2) |
| Pakpour et al., 2011b [62] | Reliability and validation study | HD patients from dialysis centres. | Iran | 144 (42.4) | 54.6(+ 11.4) | Dialysis:  30.9 (± 22.2) |
| Park et al., 2007 [45] | Reliability and validation study | HD and CAPD patients at a university dialysis centre. | Korea | 164 (41.5) | Men:  54.0 (+ 12.7)  Women:  54.3 (+ 13.6) | Dialysis:  56.7 (+ 61.3) |
| Perneger 2003 [90] | Cross-sectional study of ESRD patients | ESRD patients treated by in-centre HD identified from patient lists provided by 4 dialysis units. | Geneva canton, Switzerland | 83 (37.0) | 60.0 (+15.5) | Not reported |
| Rao et al., 2000 [59] | Developmental and validation study of subscales of KDQOL instrument. | Secondary analysis of data collected for another study of HD patients. | USA | 165 (52.0) | 20 - 65+ | Not reported |
| Rebollo et al., 2003 [79] | Cross-cultural adaptation and validation study. | ESRD patients undergoing chronic HD or PD on the Tx waiting list were recruited at the moment of the pre-transplant examination. | Spain | 54 (32.0) | Median age: 51.0  Interc. Range:  (38.0 – 57.0) | Not reported |
| Ricardo et al., 2013 [34] | Reliability and validation study | Individuals with mild-to-moderate CKD (CKD stages not clearly defined) enrolled in the Chronic Renal Insufficiency Cohort (CRIC) Study and the Hispanic CRIC (HCRIC) Study. | USA | 829 (40.0) | 57.0 (+11.6) | Not reported |
| Stavem and Ganss 2006 [72] | Reliability and validation study | Tx recipients of a hospital nephrology outpatient clinic. | Norway | 53 (42.0) | 57.5 (+13.2) | Tx median (range):  4.4 (0.4 - 29.1) years |
| Suet-Ching 2001 [49] | Development and validation study | Patients from dialysis centres. | Hong Kong, China | 164 (51.0) | 41 - 70  (70% total sample) | Not reported |
| Tao et al., 2014 [38] | Reliability and validation study | Patients with mild-to-severe CKD (CKD stages 1 – 4) were recruited from the renal wards and outpatient dialysis clinics of a tertiary hospital. | Mainland China | 103 (44.8) | 47.6 (14.2) | Dialysis:  45.9 (+41.4) |
| Thaweethamcharoen et al., 2013 [41] | Reliability and validation study | HD and PD patients were recruited from hospital outpatient clinics. | Thailand | HD 167 (52.1)  PD 62 (49.1) | HD:  57.5 (+15.1)  PD:  67.6 (+11.5) | HD:  7.4 (+5.4) years  PD:  1.9 (+1.2) years |
| Vasilieva 2007 [95] | Cross-cultural adaptation and validation study | Chronic HD patients | Russia | 91 (42.0) | 50.2 (+ 12.8) | Not reported |
| Weisbord et al., 2004 [52] | Developmental study | This study is part of a larger study of symptoms in ESRD. HD patients were selected from a dialysis unit. | USA | 20 (5)  (Test re-test) | Not reported | Not reported |
| Wu et al., 2001 [50] | Developmental study | This instrument was developed as part of CHOICE study, for patients with ESRD to complement the SF-36.  A random sample of HD and PD patients attending 4 dialysis centres. | USA | 928 (46.0)  HD: 694  PD: 234 | 58 | Dialysis< 1 year |
| Yang 2013 [40] | Validation study | Secondary data analysis study of data from a cross-sectional survey of HD patients treated at a dialysis centre. | Singapore | 394 (44.2) | 52.4 (+11.7) | Dialysis:  69.0 (+50.6) |
| Yang et al., 2006 [63] | Development and validation study | Patients undergoing regular HD at the dialysis centres of 10 regional hospitals or outpatient clinics. | Taiwan | 249 (49.0) | 62% < 50 years old;  20% < 40 years old. | Not reported |
| Yildirim et al., 2007 [91] | Cultural adaptation and validation study | Patients with ESRD randomly selected from dialysis units of 3 educational and research hospitals. | Turkey | 82 (65.0) | 51.0 (+ 12.0) | Dialysis: median 3 years. |
| Zengin et al., 2014 [60] | Cross-sectional study | Patients receiving treatment in 3 different HD centres. | Turkey | 172 (40.7) | 49.7 (± 14.7) | HD: 7.5 years |

D - Dialysis; HD - Hemodialysis; Tx - Renal transplant; ESRD – End stage Renal Disease; CKD – Chronic Kidney Disease; CRD – Chronic Renal Disease; CRF – Chronic Renal Failure; SD - Standard deviation; CAPD - Continuous ambulatory peritoneal dialysis; QOL – Quality of life.
