# Supplementary material for: Measurement properties of patient-reported outcome measures (PROMs) used in adult patients with chronic kidney disease: A systematic review
Source: PLoS One. 2017 Jun 21;12(6):e0179733. doi: 10.1371/journal.pone.0179733 (PMC5479575; doi:10.1371/journal.pone.0179733)
Supplement: S3 Table — (DOCX) [file pone.0179733.s006.docx]

| **S3 Table. Characteristics of included PROMs** | | | | | | |
| --- | --- | --- | --- | --- | --- | --- |
| **Instrument name** | **Study ID** | **Study language(s)** | **Summary of instrument** | **Scoring method** | **Mode of administration** | **Recall period** |
| Agarwal (unnamed) | Agarwal | English | 37-item instrument  Mental and physical domains | 0 - 100 scale (worst to best) | Self-administered | 4 weeks |
| Chinese Dialysis Quality of Life Scale (CDQOL) | Suet-Ching | Cantonese | 29-item instrument | 5-point Likert scale | Self-administered | Not reported |
| CHOICE Health Experience Questionnaire (CHEQ) | Wu  Aiyasanon | English  Thai | 83-item instrument.  8 domains from SF36 (36 items) with 7 supplementary items for the CHEQ.  13 specific domains (38 items)  2 person-specific quality of life items. | Responses are transformed by simple linear regression to a scale of 0 - 100. | Self-administered | 4 weeks |
| CKD Symptom Burden Index (CKD-SBI) | Almutary | Arabic | 32-item instrument.  CKD-SBI is a modification of the DSI for use in CKD stages IV and V.  The CKD-SBI has 4 symptom dimensions. | The prevalence scale uses a Yes/No method. The range of scores is 0-32. A higher overall score indicates a higher symptom occurrence.  Distress, severity and frequency are rated using a 0 to 10 scale. A maximum total score for each of these scales is 320 (range 0-320). Higher scores indicate the higher symptom burden. | Self-reported | Extended from 1 week in the original DSI to 4 weeks to reflect the chronicity of CKD symptoms |
| Dialysis Symptom Index (DSI) | Weisbord  Onsoz | English  Turkish | 30-item instrument | A 5-point Likert scale.  Two scores are obtained. An overall symptom burden score and a total symptom severity score | Self or interviewer administered | Past week |
| End stage Renal Disease Symptom Checklist - Transplant Module (ESRD-SCL) | Franke  Ortega  Stavem | German  Spanish  Norwegian | 43-item instrument.  6 dimensions. | 5-point Likert scale.  A high score indicates a worse HRQOL. Overall scores range from 0 - 100. | Interviewer and self-administered | Not reported |
| EuroQol EQ-5D | Cleemput | Dutch,  French | The EQ-5D has a self-classifier and a visual analogue scale (VAS) which patients can use to value their current health state.  The self-classifier includes 5 dimensions and each dimension has 3 levels of severity (no problems, some problems, and severe problems). | Using the EQ-5D it is possible to describe 243 different health states. Each of these health states corresponds with a single index measure between 0 (dead) and 1 (perfect health), obtained from the preferences of the general public. | Self-report | Not reported |
| Gastrointestinal Symptom Rating Scale (GSRS) | Kleinman | German,  English | 15-item instrument.  Covers important GI symptoms that may occur with various immunosuppressive therapies.  There are 5 subscales | Each subscale gives an average score ranging from 1 (no discomfort) to 7 (very severe discomfort).  Higher scores indicate worse impact | Self-administered | Not reported |
| Gastrointestinal Quality of Life Index (GIQLI) | Kleinman | German,  English | 36-item instrument.  Focuses on the impact GI complaints on an individual’s HRQOL.  There are 5 subscales: | Subscale scores range from 0 to 4. A total score ranging from 0–144) can be obtained.  Higher scores indicate better HRQOL. | Self-administered | Not reported |
| Kidney Disease Questionnaire (KDQ) | Alvarez-Ude  Laupacis | Spanish  English | 26-item instrument.  5 dimensions  Note that the physical symptom dimension is patient-specific, thus the symptoms most important to individual patients are identified and used. | 7-point Likert scale. | Interviewer | 2 weeks |
| Kidney Disease Quality of Life-36 questionnaire (KDQOL-36) | Chao  Chow  Mateti  Ricardo  Tao  T'charoen  Yang 2013 | Taiwanese  Cantonese  Kannada  Spanish  English  Mandarin  Thai  English | 36-item instrument.  3 specific scales from the KDQOL-SF™v1.3.  Summary scales from generic SF-12. | The raw scores are transformed linearly to a range of 0 to 100. Higher scores indicate better HRQOL  The scores for the PCS and MCS are converted to T-scores with a mean of 50 and a standard deviation of 10. | Self, proxy and interviewer administered | Not reported |
| Kidney Disease Quality of Life-Short Form questionnaire (KDQOL-SF™) | Abd ElHafeez  Barotfi  Bataclan  Boini  Bouidida  Cheung  Duarte  Fardinmehr  Green  Hays  Joshi  Klersy  Kontodimopoulos  Kontodimopoulos  Korevaar  Malindretos  Molsted  Moreira  Pakpour  Park  Perneger  Vasilieva  Yildirim | Arabic  Hungarian  Filipino  French  Moroccan  Chinese  Portuguese  Persian  Japanese  English  English  Italian  Greek  Greek  Dutch  Greek  Danish  Portuguese  Farsi  Korean  French  Russian  Turkish | 80-item instrument.  Derived from KDQOL-SF. Version 1.3 differs from the 1.2 version only by the addition of a screening question about sexual activity.  8 generic (SF-36) dimensions, 8 disease-specific dimensions and 3 additional dimensions | Scores are calculated so that each domain has a potential range from 0 to 100. Higher scores indicate better HRQOL | Self or interviewer administered | 4 weeks |
| Kidney Disease Quality of Life - KDQOL (Dialysis version) | Hays 1994 | English | 134-item instrument.  KDQOL consists of SF36 generic core with 8 domains, 11 kidney disease targeted scales and an item that assesses change in health over a year (overall health rating). | Scores transformed linearly into 0 - 100 point scale.  Higher scores indicate better HRQOL. | Self or interviewer administered | Last 30 days |
| Kidney Disease Quality of Life - KDQOL (Modified) | Rao | English | 55 items from S/P and effects of KD.  Using affinity mapping, 11 subscales were identified while 4 items were ungrouped. | 0 to 100 scale. | Not reported | Not reported |
| Kidney Transplant Questionnaire (KTQ) | Chisholm-Burns  Laupacis  Niu  Rebollo | English  English  Chinese  Spanish | 25-item disease-specific measure, 5 domains.  The physical symptom domain is patient-specific, thus the symptoms most important to individual patients are identified and used. | For each domain, an average score from 1 to 7 is calculated. | Self or interviewer administered | 2 weeks |
| Modified Edmonton Symptom Assessment System (ESAS) | Davison a & b | English | 10 symptom specific items (9 original items) | 10 visual analogue scales with superimposed 0-10 scale. Total score range from 0 to 100. | Self-completed | Not reported |
| Modified Transplant Symptom Occurrence and Symptom Distress Scale (MTSOSD) | Moons | Dutch | 29-item instrument.  20 items relate to the symptom occurrence domain.  9 items relate to symptom distress domain. | Vertical scaling for symptom occurrence and a horizontal technique for symptom distress.  5-point scale (0-4) | Self-reported | Not reported |
| Nottingham Health Profile (NHP) | Badia  Zengin | Spanish  Turkish | 38 yes/no questions.  Part 1 has 6 dimensions while Part 2 is optional. | Each dimension is scored by multiplying the total positive answers with 100 and dividing the result by the number of items.  The NHP scores range between 0 (good health status) and 100 (poor health status). | Self and interviewer administered | Not reported |
| Quality of Life Index 3.0 | Dehesh  Ferrans  Halabi  Korkut | Persian  English  Arabic  Turkish | 68-items in total.  Instrument is divided into 2 sections: One section measures satisfaction with various domains of life, while the second measures the importance of the domain to the individual.  Three additional items relative to dialysis treatment were added to each section. | Ratings are made on a scale of 1 to 6 for both parts. Scores are calculated by weighing each satisfaction response with its paired importance response.  The total QOL score and the four subscale scores range between 0 and 30. | Self or interviewer administered | Not reported |
| ReTransQoL (RTQ) v1 | Beauger  Gentile | French  French | 45-item instrument, 5 dimensions | Questions are equally weighted. The score for each individual is obtained by computing each item's mean score within every dimension. All dimensions are linearly transformed to a 0–100 scale. A score of 100 indicates the highest QOL. | Self-administered | Not reported |
| ReTransQoL (RTQ) v2 | Beauger | French | 32-item instrument, 5 dimensions. | Questions are equally weighted. The score for each individual is obtained by computing each item's mean score within every dimension. All dimensions are linearly transformed to a 0–100 scale. A score of 100 indicates the highest QOL. | Self-administered | Not reported |
| Short Form-12 (SF-12) | Pakpour (b) | Persian | 12 items (questions) and 8 scales. These 8 scales can also be computed into 2 distinct clusters of PCS-12 and MCS-12 | Not reported | Interviewer | Not reported |
| Short Form-36 (SF-36) | Feurer  Mingardi | English  Italian | 36-item instrument, 8 scales | After standardizing the 8 scales to z-scores based on means and standard deviations for the general US population, aggregate physical and mental component summary scales are calculated by adding the eight differentially weighted scales.  Finally, each component score is transformed to a normalized T-score with a mean of 50 and a standard deviation of 10. | Self-reporting  Administration by a trained interviewer in person or over the telephone | Standard (4- week) version or Acute (1-week) version |
| WHOQOL-BREF (Dialysis module) | Yang 2006 | Taiwanese | 32-item instrument.  The core WHOQOL-BREF (TW) has 4 domains.  4 HD specific items incorporated into the existing domains. 2 global items. | 5-point Likert scale.  The score for each domain ranges from 4 to 20. This is calculated by multiplying the average scores for all items in the domain by 4. | Self or interviewer administered | Recent month |
| Modified Time Trade-Off (TTO) | Churchill 1987 | English | Utility measure | The lower value of 0 represents an extremely low QOL in those patients for whom death would be as preferable as their present state of health while the upper value of 1 represents a very high QOL in those patients whose present health state would be equivalent to their perception of perfect health. | Interviewer | 2 – 3 weeks |
